# Viscoelastic parameterization of human skin cells characterize material behavior at multiple timescales

**DOI:** 10.1038/s42003-021-02959-5

**Published:** 2022-01-11

**Authors:** Cameron H. Parvini, Alexander X. Cartagena-Rivera, Santiago D. Solares

**Affiliations:** 1 Department of Mechanical and Aerospace Engineering, The George Washington University School of Engineering and Applied Science, Washington, DC USA; 2 Section on Mechanobiology, National Institute of Biomedical Imaging and Bioengineering, National Institutes of Health, Bethesda, MD USA

**Keywords:** Melanoma, Computational biophysics, Nanoscale biophysics

## Abstract

Countless biophysical studies have sought distinct markers in the cellular mechanical response that could be linked to morphogenesis, homeostasis, and disease. Here, an iterative-fitting methodology visualizes the time-dependent viscoelastic behavior of human skin cells under physiologically relevant conditions. Past investigations often involved parameterizing elastic relationships and assuming purely Hertzian contact mechanics, which fails to properly account for the rich temporal information available. We demonstrate the performance superiority of the proposed iterative viscoelastic characterization method over standard open-search approaches. Our viscoelastic measurements revealed that 2D adherent metastatic melanoma cells exhibit reduced elasticity compared to their normal counterparts—melanocytes and fibroblasts, and are significantly less viscous than fibroblasts over timescales spanning three orders of magnitude. The measured loss angle indicates clear differential viscoelastic responses across multiple timescales between the measured cells. This method provides insight into the complex viscoelastic behavior of metastatic melanoma cells relevant to better understanding cancer metastasis and aggression.

## Abstract (web summary)

Parvini, Cartagena and Solares introduce an iterative viscoelastic approach based on the generalized Maxwell and Kelvin-Voigt models. The results showed that metastatic melanoma cells had lower elasticity than normal fibroblasts and melanoma cells were less viscous than the fibroblasts over a large frequency range, enhancing the understanding of cellular responses at different frequencies.

## Introduction

Current state-of-the-art mechanobiological applications involve testing samples that are soft, viscous, and/or polymeric in nature^1^. Understanding the mechanical character of these materials at the nanoscale is especially important in biological studies^2–4^. Whether the goal is characterizing biophysical behavior in human lung epithelial cells^5^, describing the various microrheological phases that isolated adherent cells generally exhibit^6^, investigating why cancer cells apparently soften during disease progression^7^, or investigating why human cardiac cells showcase both shear-force fluidization and strain-stiffening while beating (despite appearing contradictory)^8^, using an appropriate analytical treatment for soft sample mechanics is critical to correctly describing complex biological processes.

Cancers are a group of diseases that possess a common characteristic of leading to the development of transformed cells exhibiting a rapid and uncontrollable burst of growth and proliferation, which in turn leads to the formation of a solid tumor^9^. Metastatic cancer is an aggressive disease, characterized by its high level of mutational burden, resistance to traditional chemotherapies, and rapid metastasis dissemination^9^. To successfully metastasize, cancerous cells must migrate away from the primary tumor, invade the surrounding tissue, extravasate through the circulatory system, and intravasate through the vasculature to colonize a new tissue^10,11^. While cancer cells can use a variety of migration strategies, metastatic cells are subjected to the stringent physical constraints of the extracellular matrix (ECM)^11–13^. Thus, to successfully pass-through micron sized gaps in the ECM, the cell actomyosin cortex and its nucleus (the largest and stiffest organelle in the cell) must be deformed to a high extent (over 50−80% of its original size), thus experiencing large external mechanical stresses^12–14^. Therefore, the mechanical properties of cancerous cells, including melanomas, are critical for the successful survival, migration, and colonization of tissues.

Recent work has shown that the intracellular mechanical properties are mostly controlled by the cytoskeleton, a network of intermediate filaments, microtubules, and filamentous actin^15^. Cells are often considered to be soft biomaterials that behave as complex viscoelastic fluids in terms of their response to external mechanical stresses^16,17^. By applying atomic force microscopy (AFM), cancer cells have been shown to be more deformable than their normal untransformed counterparts^7,18^, potentially due to modifications in cytoskeletal organization, possibly aiding them with upregulated migration through a structurally and mechanically complex ECM^19–21^. However, most of the current cell mechanics research has relied upon continuum mechanics models which assume cells exhibit purely elastic behavior^22^; this approach has been frequently applied when characterizing cancerous cells^7,23–29^. While this methodology may be useful under very specific conditions, this linear elastic treatment fails to capture and properly account for the rich temporal information available in soft sample mechanical behavior datasets. For example, in applications using AFM to evaluate cell properties, the difference between assuming linear-elastic sample behavior versus more complex viscoelastic mechanics can be significant^3,30–33^. While there have been a few investigations into linear viscoelastic assumptions for soft samples^34–38^, these implementations rarely account for more than two discrete stiffnesses and one retardation time. For cellular action taking place over a broad duration, a single retardation time could not simultaneously account for both small- and large-timescale mechanical responses. In this case, it is necessary to approximate physical action using a model that successfully delineates between timescales, such that there still remains a need to develop generalized viscoelastic models for soft cells.

Using a modified approach outlined in this paper, we build upon a previous open-search viscoelastic parameter extraction methodology^31^ to parameterize biological samples with a dynamic number of retardation times, and search for new mechanical information that emerges at multiple timescales. One clear application is for use on cancerous cells, where directly tracking the effects of the viscoelastic harmonic response as a function of frequency could uncover new mechanical signatures that are useful for the early detection of common cancers, such as metastatic melanoma. Characterizing the mechanical response that occurs over short timescales (at seconds timescales) could also provide significant insight into individual cellular processes, such as filamentous actin cortex cytoskeleton remodeling^39^, turnover of actin filament crosslinkers^40^, myosin motor fast contractility^41^, microtubule assembly or disassembly^42^, and vesicle trafficking^43^ to name several. These single-cell biological processes are essential for cellular tissue functions including cell and tissue homeostasis, development, and disease progression including cancer metastasis. The method presented here also reduces the computational overhead for each individual parameter fit by limiting the associated parameter space. This decreases the number of iterations necessary to obtain each unique mathematical series describing the material, leading to comparable overall parameterization times and less susceptibility to local minima versus the previous methodology. While this iterative approach still allows for further optimization, it provides a marked improvement over the open-search methodology and has been applied successfully for soft samples.

This study begins by introducing the iterative method used to improve the accuracy of the fitting functions, showing that the performance of the approach is far superior and more accurate than standard open-search methods. We then utilize the method to characterize the viscoelastic behavior at multiple retardation times of 2D adherent human metastatic melanoma cells and compare them with their normal counterparts, primary epidermal melanocytes, and fibroblasts. Our viscoelastic measurements reveal that 2D adherent metastatic melanoma cells are less elastic and moderately viscous over the relaxation timescale studied when compared to melanocytes and fibroblasts, whereas fibroblasts are the stiffest and more viscous. Interestingly, melanocytes have intermediate stiffness, while they are the least viscous. Altogether, the study provides a general understanding of the complex viscoelastic behavior of living metastatic melanoma, which is relevant to better understanding metastasis and aggression.

## Results

### Improved characterization of cells viscoelastic behavior by parameterization approach

**Fig. 1:**
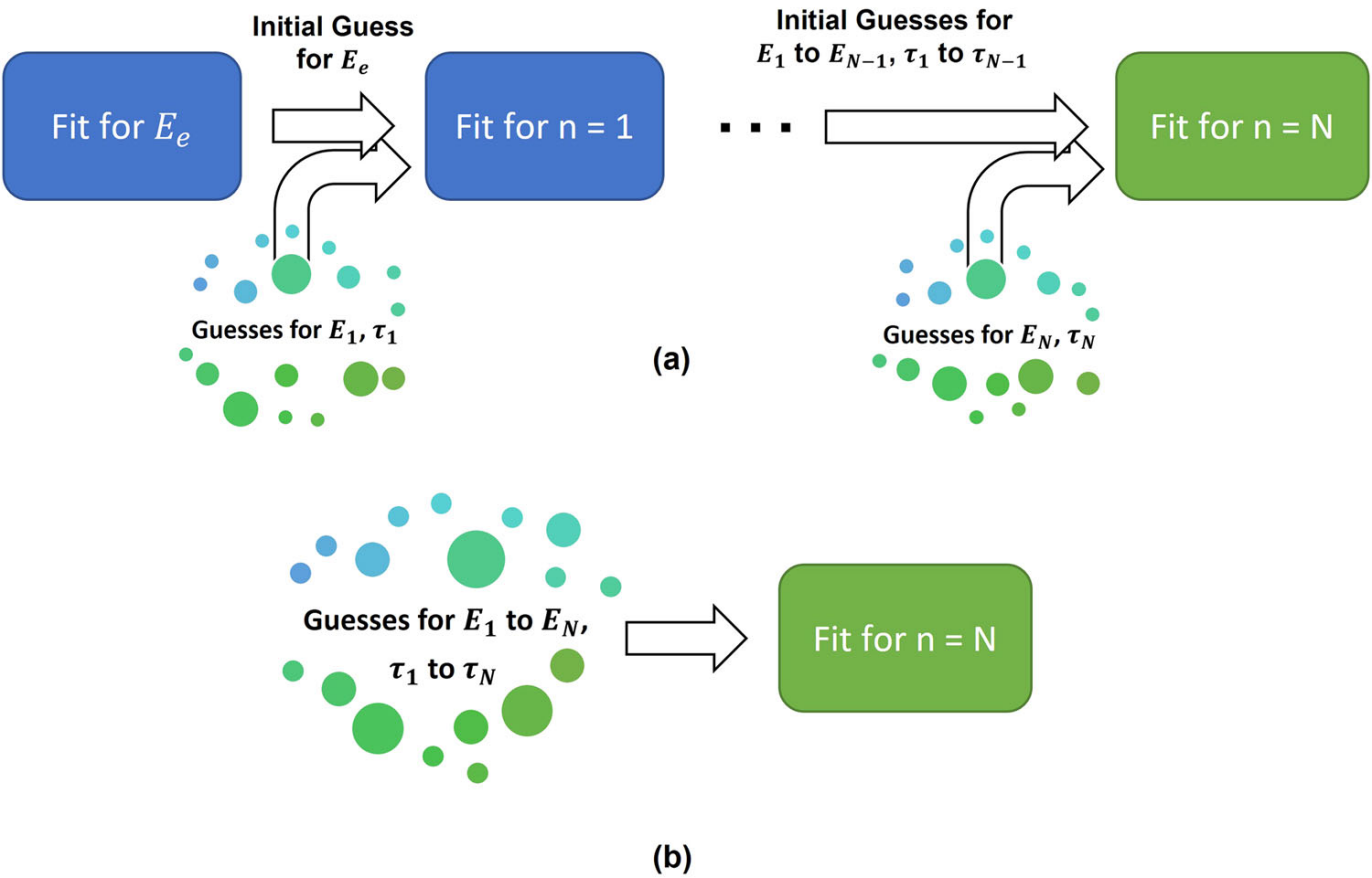
Flowchart comparison for the iterative and open-search methodologies. The process visualization for the iterative-fitting (**a**) and open-search (**b**) methodologies are shown here, using increasingly more complex configurations within a generalized viscoelastic model (see Fig. 2f). As depicted, the iterative-fitting methodology involves slowly expanding the desired generalized viscoelastic model while iteratively re-parameterizing each new configuration. The “optimal” parameter set for a given configuration is used as the initial guess in the next fitting attempt. In this scheme, a random starting point is generated only for the new parameters. In the open-search methodology, there is no a priori information provided to the parameterization process, aside from the valid parameter bounds and a random initial guess for each parameter in the model. The variable “*N*” represents the *N*th element introduced into the generalized viscoelastic model.

One shortcoming of using an open-search methodology^44^ to parametrize a viscoelastic material using a discrete generalized model is the inability to pinpoint the contribution of each viscoelastic element in the model to the overall mechanical response. This is due primarily to the fact that model elements are not individually treated during the fitting process—all parameters are estimated simultaneously using a nonlinear least-squares regression approach (Fig. 1b). The associated parameter space for each fit is large, which can easily lead to improper coupling of different elements. For example, in the case of a multi-element material model, the fitting algorithm could artificially increase the stiffness of one particular element to account for an improperly tuned characteristic time in another element. The algorithm would therefore skew the stiffnesses of both elements and fail to correct the error in the characteristic times, while still providing an apparently good fit to the experimental dataset. This shortcoming of the open-search method may not be critical when the user only seeks a close approximation, but can be detrimental to the quality of the final fit, and may require refinement at an additional, unnecessary cost. Aside from these associated computational penalties, the range of values that provide an adequate fit to the data can have drastically different implied mechanical properties, which in turn complicates and adds uncertainty to the analysis of the sample response. These ambiguities seem to be exacerbated in the case of soft biological samples. By contrast, the iterative-search methodology (Fig. 1a) introduces new elements in turn and utilizes the previous optimization results as an initial estimate for those parameters in a new, more complex model.

**Fig. 2:**
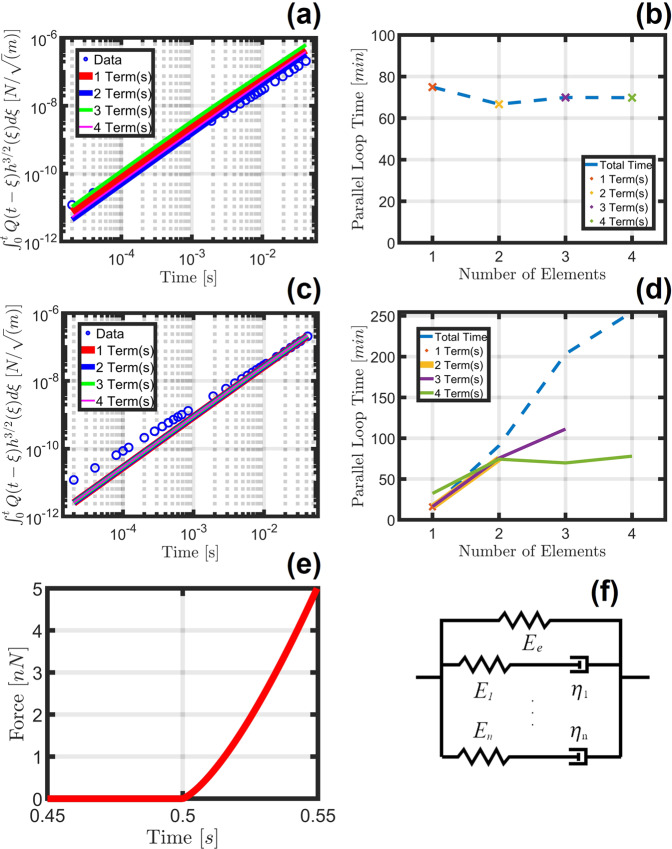
Performance comparison for the iterative and open-search methodologies. The fit performance and computational requirements for the Open Parameter Search method (**a** and **b**), and the Iterative Term Introduction method (**c** and **d**) are shown, in addition to the original force curve (**e**) generated using the Generalized Maxwell Viscoelastic model (**f**) with parameters provided in Supplementary Table 1 and simulated according to the methods described in Supplementary Discussion 5. The dashed blue lines in **b** and **d** represent the total time necessary to acquire a set of best-fit parameters for a model with the number of elements indicated—i.e., for four elements, the Iterative Term Introduction method would require just over 250 min to run the fitting for terms 1−4, while the open parameter search method needs only the time indicated for the 4-term fit (approximately 70 min). The fitting time for the iterative approach using 1 term is lower than the corresponding fit for the open parameter search method because in the former the elastic term is first fit independently, and then fed forward to the 1-term parameterization attempt. The standard error for each model configuration is provided in Supplementary Table 2. Note that fit quality (**a** and **c**) has been visualized using the Generalized Maxwell Viscoelastic model convolution integral of the fit (${\int }_{0}^{t}Q\left(t-\zeta \right)* {\left\{h\left(\zeta \right)\right\}}^{\frac{3}{2}}{{{{{\rm{d}}}}}}\zeta$) against the normalized dataset ($\frac{3\left(1-\nu \right)}{8\sqrt{R}}F\left(t\right)$).

To evaluate the efficacy of both the open-search and iterative approaches, the elapsed time was tracked while a simulated AFM quasi-static force curve was used to parameterize a generalized viscoelastic model. The results, in addition to the fit quality as a function of the number of terms introduced, are shown in Fig. 2 for both approaches. For this evaluation case, 500 fitting iterations were run for each unique series, with up to four timescales (each requiring one separate term in the model), beginning with an initial timescale on the order of 10^−4^ seconds and using increasing timescales for each new model term. The associated errors for each model configuration have been included in Supplementary Table 2, which indicates that the iterative approach resulted in standard errors roughly one order of magnitude smaller than the open-search.

The elapsed fitting time (Fig. 2b, d) clearly shows that the iterative approach required a longer time to fit an equivalent number of terms when compared with the open-search approach. This is expected, since the iterative method requires separate calculations for each number of elements leading up to the desired final quantity. Although the overall time requirement was larger, the fitting time of each term in the iterative case (considered individually) was of similar order of magnitude as for the open-search case. In addition, the nearly overlapping lines in the iterative case showcase its remarkably stable performance (Fig. 2c), in contrast with the relatively small but non-monotonic variations in the open-search (Fig. 2a). This observed instability for the open-search method can lead to degraded performance for experimental data sets exhibiting significant noise contributions. Overall, when evaluating the performance and fitting time requirement, the iterative approach methodology required a moderate increase in time while providing a more repeatable performance. In addition, when the user is interested in evaluating the results as a function of the number of terms introduced (this is recommended in order to avoid “overfitting” the data, since the required number of viscoelastic model elements is not known a priori), the iterative method can provide that information faster relative to the open-search method. For example, summation of all points in Fig. 2b reveals that the total time to obtain this information for the open-search method (~280 min) is larger than the “Cumulative Time” in Fig. 2d (~250 min) for a four-term fit using the iterative method. If more elements are desired, the difference in time required to obtain this information would become increasingly different for the two approaches.

### Minimal parameterization terms required for cell viscoelastic characterization

The improved stability of the iterative parameter extraction methodology enables its application to samples that were previously difficult to analyze and differentiate, making it especially relevant for biological and other soft samples. To implement this approach, AFM force curves were collected from 2D adherent human metastatic melanoma cells, as well as their normal counterparts, primary epidermal melanocytes and fibroblasts at the nuclear region (Supplementary Fig. 1).

The critical point of merit for a parameterized model’s performance is how closely it reproduces the normalized dataset. In this case, indentation depth and force have been experimentally measured with AFM and scaled according to one of the following equations:

1 $${\left\{h\left(t\right)\right\}}^{\frac{3}{2}}=\,\frac{3\left(1-\nu \right)}{8\sqrt{R}}\,\int_{0}^{t}U\left(t-\zeta \right)* F(\zeta ){{{{{\rm{d}}}}}}\zeta$$

2 $$F(t)=\,\frac{8\sqrt{R}}{3\left(1-\nu \right)}\,\int_{0}^{t}Q\left(t-\zeta \right)* {\left\{h\left(\zeta \right)\right\}}^{\frac{3}{2}}{{{{{\rm{d}}}}}}\zeta$$

Here, the measured indentation (*h*) and force (*F*) have been convolved with the model-specific viscoelastic Relaxance (*Q*) or Retardance (*U*), and then scaled by the tip radius (*R*) and Poisson ratio ($\nu$), in accordance with the well-known Lee and Radok spherical indentation framework^45^ (Supplementary Fig. 2). Using each compliance- (Eq. 1) or stiffness-based (Eq. 2) model description, the convolution term has been calculated using the best-fit parameters and the repulsive (i.e., compressive force application) portion of the indentation force curve. For a full description of the analytical methods applied when deriving the material Relaxance (*Q*) or Retardance (*U*), see Supplementary Discussions 1−4 and 8, which also provides detailed guidelines for the choice of a viscoelastic material model. The optimal fit quality is visualized here for each unique cell type within the stiffness-based Generalized Maxwell Model description (Eq. 2)^46^. Calculations were also performed using the compliance-based Generalized Kelvin−Voigt Model (Eq. 1) which yielded similar results. This behavior is expected since both models are physically equivalent^46^. Note that fit quality has been visualized throughout this manuscript using the viscoelastic model convolution integral of the fit (${\int }_{0}^{t}Q\left(t-\zeta \right)* {\left\{h\left(\zeta \right)\right\}}^{\frac{3}{2}}{{{{{\rm{d}}}}}}\zeta$) against the normalized dataset ($\frac{3\left(1-\nu \right)}{8\sqrt{R}}F\left(t\right)$)—see Eqs. 1 and  2. This approach separates the terms which are dependent upon the model parameters (via the Relaxance and Retardance) from the quantities that are known or observed from the experiment.

**Fig. 3:**
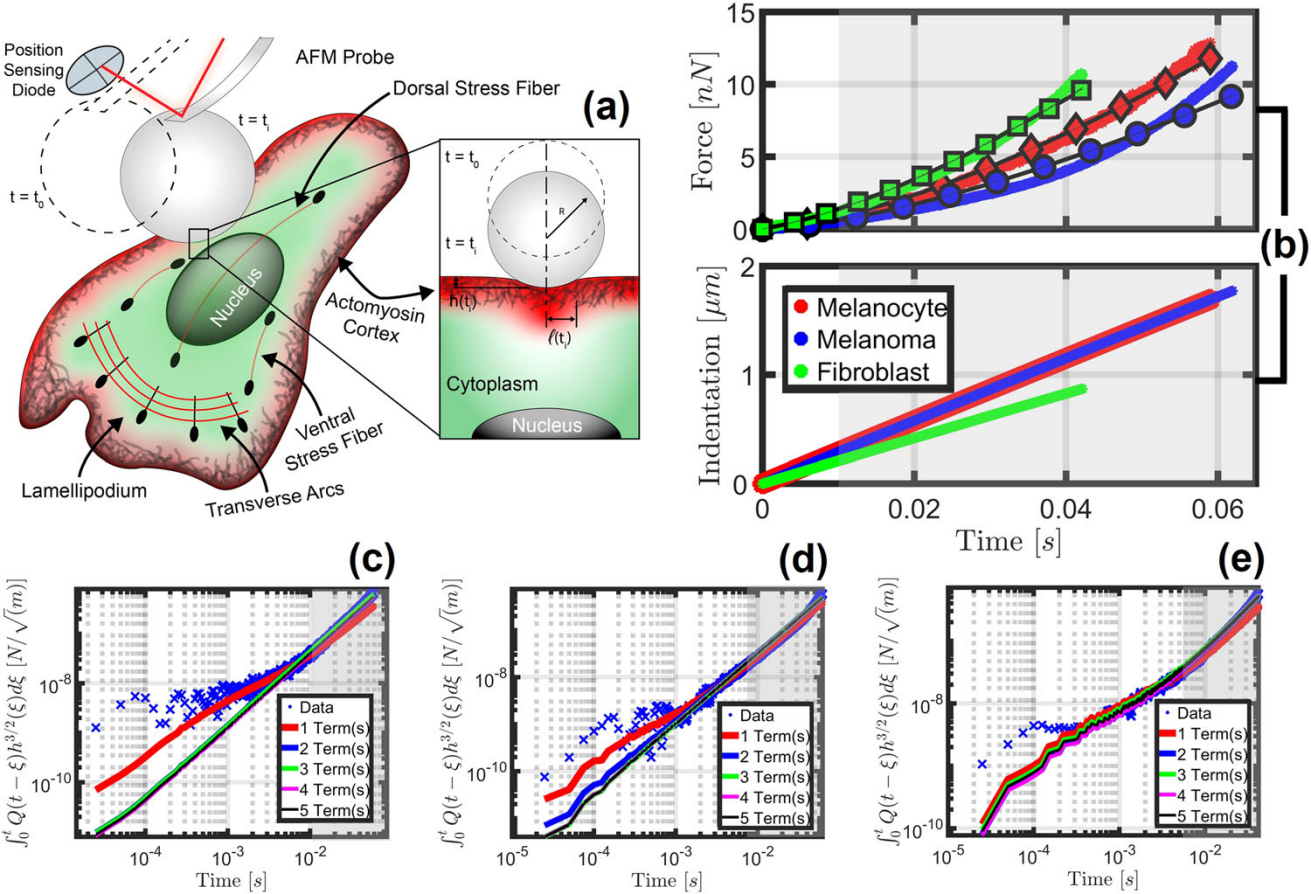
Experiment rendering and representative example datasets used for viscoelastic parameterization. This figure showcases the model performance vs. action integral data for the 2D adherent human skin cell lines under study. The experiment configuration is rendered in (**a**). Note that *h*(*t*_*i*_) is the final indentation depth and *l*(*t*_*i*_) the final contact radius. The force curves reconstructed using the optimal parameter sets for each adherent cell type are provided in (**b**), where the thick colored lines represent the observed data and the thinner, marked lines represent the optimal model estimations. For subfigure **b** the melanocyte model was fit using two viscoelastic elements, the melanoma model using three elements, and the fibroblast model also used two elements. The experimental data collected through the AFM force curves has also been visualized in terms of an action integral in log-spaced form as scattered markers (“Data”), and the corresponding colored lines show the action integral predicted by the model’s approximation of that dataset for a varying number of terms for melanocytes (**c**), melanoma (**d**), and fibroblasts (**e**), respectively. These subfigures are the basis upon which the “optimal” parameter sets are determined; the lowest number of terms that most accurately represent the input data has been selected in each case. In some cases, the first order of magnitude was difficult to approximate (as is evident in (**c**−**e**), especially for melanoma), due in part to data acquisition frequency limitations and the cost of performing numerical convolutions. Gray shaded regions represent the same temporal regions in (**b**−**e**) and have been included to showcase why subtle changes at longer timescales in (**c**−**e**) are critical to the quality of fit in the timescales visible in (**b**) and are given additional preference over high-quality fits at shorter times. This figure showcases results for the Generalized Maxwell Model in the Lee and Radok framework. Panels **b**−**e** show the results for a single representative force curve taken at the nuclear region of the cell; this process was repeated for between 70 and 193 AFM force curves from each cell type. Note that the sparse markers for the model estimations included in the force plot (**b**) are deliberately periodically spaced for representation purposes to differentiate the model estimation from the dataset without completely obscuring the latter—there are an equivalent number of model and AFM observable datapoints. The standard error for each model configuration in (**c**−**e**) is provided in Supplementary Table 2. In addition, the fit quality **c**−**e** has been visualized using the Generalized Maxwell viscoelastic model convolution integral of the fit (${\int }_{0}^{t}Q\left(t-\zeta \right)* {\left\{h\left(\zeta \right)\right\}}^{\frac{3}{2}}{{{{{\rm{d}}}}}}\zeta$) against the normalized dataset ($\frac{3\left(1-\nu \right)}{8\sqrt{R}}F\left(t\right)$).

As is evident from Fig. 3c–e, the introduction of additional terms into the model does not always significantly alter the resulting action integral representation of the normalized datasets—in each case, the observation of subtle improvements at the longer timescales was the primary indicator of the optimal model’s configuration within the chosen viscoelastic model representation. This highlights the need to iteratively introduce elements: the qualitative effects of each element must be well understood and must provide clear benefits over simpler configurations in the frequency range of interest in order to be useful. In the cases shown, two terms were used to fit the melanocyte dataset (Fig. 3c), three elements were necessary to fit the melanoma dataset (Fig. 3d), and two terms were necessary for the fibroblast dataset (Fig. 3e). Notice that adding a second term to the melanocyte model improved the fit, however including additional terms beyond three only served to degrade the fit quality. Clearly, the inclusion of a large number of elements in the model is not always beneficial.

Overall, the fit to the observed force (Fig. 3b) indicated that the chosen parameters can be used to generate satisfactory representations of the material viscoelastic behavior for most timescales, within the leading order of magnitude. Nevertheless, it is important to point out that the linear viscoelastic model chosen was unable to fully reproduce the AFM force curves, in particular the apparent stiffening behavior observed at longer timescales (larger indentation values) in Fig. 3b. In all three cases, the experimental force curves initially rise slowly, and afterwards rise more sharply, with a functional dependence that corresponds to a slope that is greater than the slope allowed by the chosen viscoelastic model. Specifically, in an elastic spherical indentation experiment, the force is proportional to the indentation raised to the power 3/2. For a viscoelastic material represented by the Generalized Maxwell model, the exponent on the indentation will be smaller than 3/2, since the sample experiences relaxation, and this exponent can only decrease (not increase) at longer timescales. Thus, while the chosen model offers a significant improvement over the state of the art, especially over approaches relying on purely elastic approximations, the complexity in the mechanical response of biological materials may require more elaborate mechanical models, depending on the level of accuracy sought in representing their material behavior. This is due to the fact that such systems are generally heterogeneous and nonlinear, and are often characterized using geometries that are not idealized (e.g., our systems are not infinite, perfectly flat surfaces). For example, it is clear from the indentation data (Fig. 3b) that, especially for the melanocytes, the indentations were relatively large, which could be causing the stiff nucleus to manifest itself in the force curves. Alternately, a thick glycocalyx layer on the melanoma could be causing the obvious shift in slope beyond 0.02 s. While there is no confirmation of these examples in the given datasets, neither material complexity would be captured in the current approach nor other state of the art viscoelastic frameworks.

### Multiple timescales viscoelastic characterization of 2D adherent normal and cancerous human skin cells

**Fig. 4:**
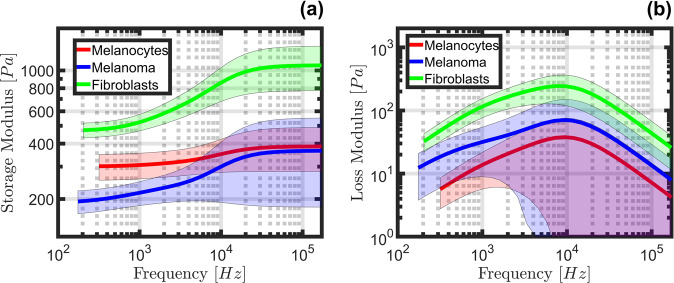
Viscoelastic harmonic quantities acquired from 2D adherent normal and cancerous skin cells. The Storage (**a**) and Loss (**b**) modulus have been calculated from the parameterized Generalized Maxwell model for 2D adherent normal and cancerous human skin cells. These viscoelastic harmonic quantities are calculated using the optimal parameter set obtained from analyzing an average of 70, 71, and 193 force curves from the melanoma, melanocyte, and fibroblast cell conditions, respectively. In total, the averaged datasets contain curves collected from 13 unique melanoma cells, 12 unique melanocytes, and 33 unique fibroblasts. Note that the number of terms in the fitted viscoelastic model series determines the number of distinct features present in the plots. The observed confidence intervals are shaded in matching colors for each model; the method for determining these bounds is outlined in the “Methods” section, and is based on the optimal parameter sets obtained using every individual curve from each cell type.

The viscoelastic harmonic functions, storage, and loss modulus, for 2D adherent normal and cancerous human skin cells are plotted in Fig. 4. These results are the figure of merit for viscoelastic analysis, which indicates how elastic or viscous the tip-sample interaction was as a function of deformation frequency (or timescale, which is the inverse of frequency). A large storage modulus indicates a strongly elastic action (increased stiffness), and similarly, a large loss modulus indicates a strongly viscous action (increased viscosity) at the corresponding frequency. It is important to note that the range of frequencies shown in Fig. 4 is not being individually excited, as with other common AFM modes based on harmonic (oscillatory) excitation^2,4^. Instead, the convolution integral used by the Lee and Radok framework implies that the AFM observables can be recreated using a series of impulses having varying magnitudes and timescales^31^. Thus, by treating each timestep as an excitation with a frequency of ${t}_{i}^{-1}$ (where ${t}_{i}$ is the current experiment time), a simple ramp input is capable of exciting mechanical action at all frequencies between the sampling rate (in this case, 50 kHz) and the inverse of the experiment length. This range of frequencies is then used with the parameterized viscoelastic model to visualize the harmonic response. For further details on calculating the viscoelastic harmonic moduli, the reader is directed to Supplementary Discussion 8.

In general, all three cell types analyzed appear soft, showing storage moduli on the order of 10^2^−10^3^ Pa. The elastic response to indentation noticeably decreases below 10 kHz for all three cell types. The largest relative change occurs for the melanoma and fibroblast datasets, which exhibit a nearly 50% reduction in elastic action between the timescales under study. Both the melanocytes and fibroblasts exhibited relatively tight confidence intervals for the storage modulus. By contrast, the melanoma dataset features a remarkably wide confidence interval at the higher frequencies. The largest viscous action was observed for the fibroblast dataset, peaking near the onset frequency of elastic stiffening (~ 10 kHz). The melanoma dataset showcased moderate viscous action, which similarly peaked near 10 kHz, but also showcased a distinct feature for frequencies below 10 kHz; the decrease in viscous action appears to temporarily diminish at a lower rate between 500 Hz and 1 kHz before declining more sharply with decreasing frequency (longer timescales). Lastly, the melanocyte dataset exhibited the lowest viscous action, peaking once again near the onset of elastic stiffening, with loss modulus values that are approximately one order of magnitude below those of its storage modulus (~10^1^ Pa as compared to ~10^2^ Pa, respectively). Interestingly, despite the apparently good fit to the force curves in Fig. 3b for melanoma and melanocytes, the confidence intervals are relatively large for the corresponding viscoelastic harmonic quantities plotted in Fig. 4 (blue and red traces), especially for the loss modulus, which may be due to factors such as variability in the overall viscoelastic behavior of the sample, noise, or mechanical nonlinearities not captured by the model. In fact, the metastatic melanoma cells used in this study are highly dynamic and migratory, therefore possibly the higher turnover of the cytoskeleton structures and translocation of the melanoma cells would impact the viscoelastic harmonic quantities. It is noteworthy to mention that the averaged indentation response for every cell line exhibits a clear peak in viscous action at nearly the same frequency, despite representing very different force curves in Fig. 3b.

**Fig. 5:**
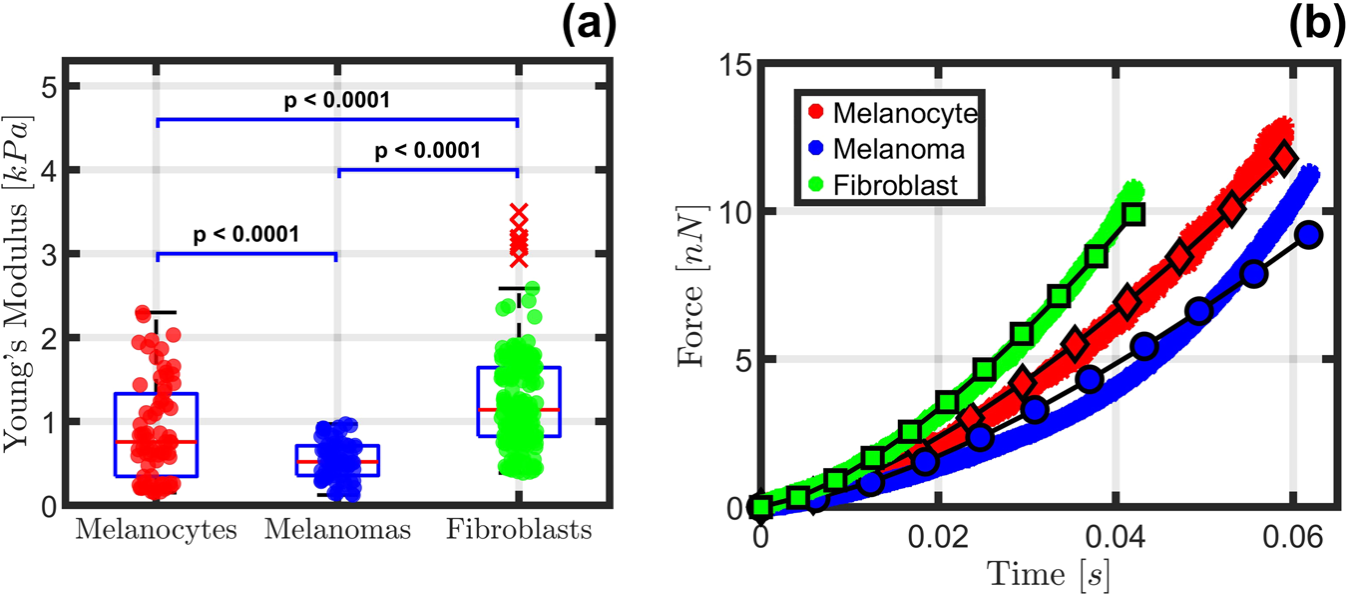
Statistical analysis and corresponding force curve estimation by parameterizing Young’s modulus for each cell type. Pseudo-Elastic (Young’s) modulus distribution (**a**) and examples illustrating the fit quality for the Pseudo-Elastic model (**b**) for the adherent human skin cell lines visualized in Fig. 3. This plot provides some insight regarding the overall stiffness for each cell type, specifically that the melanoma cells appear softer than their healthy counterparts by a noticeable margin. The red markers indicate “outlier” values, which are determined to be more than 1.5 times the interquartile range away from the upper and lower bounds of the boxes. The Fibroblasts showcase the only outliers by this definition, which can be reasonably expected because significantly more curves were acquired for that cell line. The number of cells analyzed per cell type is *n* = 12 melanocytes, *n* = 13 melanoma cells, and *n* = 33 fibroblasts. Data are represented as mean ± deviation with significant difference between cell types determined by unpaired two-tailed Student’s t-test with Welch’s correction indicated as, *P < 0.05. Note that the sparse markers for the model estimations included in (**b**) are deliberately periodically spaced for representation purposes to differentiate the model estimation from the dataset—there are an equivalent number of model and AFM observable datapoints. The standard error for each cell type is provided in Supplementary Table 2.

In addition to the results presented in Fig. 4, the traditional Hertzian spherical contact model was parameterized to provide a pseudo-elastic (Young’s) modulus for each cell type, yielding the results shown in Fig. 5 for the same datasets visualized in Figs. 3 and 4. The median Young’s modulus values are appreciably larger than the moduli plotted in Fig. 4, with the Hertz model Young’s modulus values ranging from ~600 Pa to 1.2 kPa and the viscoelastic storage modulus values ranging from ~200 Pa to 1.1 kPa. Nevertheless, the modulus values for both models were within the same order of magnitude and ranked the cell type stiffnesses in the same order. Similar to the viscoelastic model fits, the Hertzian model fits did not always properly reproduce the curvature of the experimental force curves (Fig. 5b), both over- and underestimating the forces for the short and intermediate timescales (0.1−10 ms), especially for the melanoma. In fact, the fit quality for the chosen force curves obtained with the Hertzian model seems to be comparable to the fit quality obtained with the viscoelastic models, which partly explains why the simpler elastic treatment is often assumed to be satisfactory. However, the critical shortcoming of the Hertzian approach is that it lacks the proper physics, reducing the material response to a single number that is independent of deformation rate, which makes it unable to describe the material viscous response. Generalized viscoelastic models, by contrast, incorporate the proper physics and provide a richer perspective from which material behavior at different deformation timescales can be inferred.

**Fig. 6:**
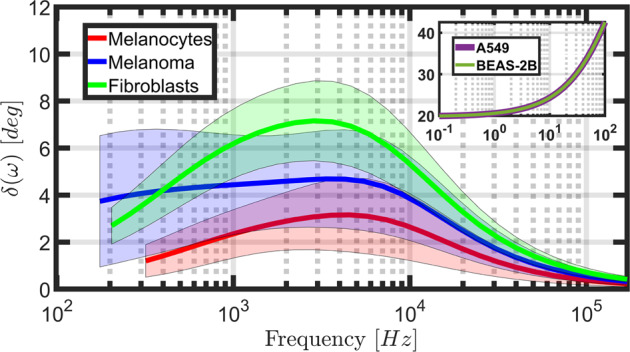
Loss angle comparison of the generalized Maxwell viscoelastic model and power law rheology (PLR) models for various adherent human skin cell types. The generalized viscoelastic models showcase non-monotonic behavior for all human skin cell lines across a wide frequency range when compared with the relatively smooth and monotonic PLR model prediction for human lung epithelial cells. Loss angles have been provided for each cell type and showcase nonlinear, non-monotonic action as a function of frequency. This is in contrast to the inset data, which was reproduced from the literature^5^ using a PLR model for human lung epithelial cells and shows a completely monotonic response with increasing frequency. While the frequency ranges for the primary axis and inset do not overlap, the PLR model is clearly lacking in frequency domain features and is merely provided to represent a typical PLR model prediction. Observed confidence intervals are shaded in matching colors for each model. When comparing all three skin cell types, the melanocyte and fibroblast models showed noticeably tighter confidence intervals than the melanoma. As with Fig. 4, these loss angle estimates were obtained by analyzing an average of 70, 71, and 193 force curves from the melanoma, melanocyte, and fibroblast cell conditions, respectively. In total, the averaged datasets contain curves collected from 13 unique melanoma cells, 12 unique melanocytes, and 33 unique fibroblasts.

In analyzing viscoelastic behaviors, it is helpful to consider not only the magnitude of the storage and loss moduli, but also the implied loss angle. This quantity, which varies between 0° and 90°, is the inverse tangent of the ratio of loss modulus to storage modulus and provides information on the *relative* magnitude of viscous to elastic action present within the dataset at different timescales. A higher loss angle indicates proportionally greater viscous action, whereas a smaller value indicates proportionally greater elastic action. The loss angle for the three distributions shown in Fig. 4 has been plotted in Fig. 6 together with results for human lung epithelial cells from the literature^5^. The lung epithelial cell results are based on fitting the mechanical behavior measured with AFM to a power-law rheology (PLR) model, for which the response of the material must follow a specific monotonic trend defined by the power-law parameters. This is in contrast to our use of generalized models, which do not constrain the response at a given timescale to exhibit any specific trend with respect to the response at other timescales. From the loss angle plots in Fig. 6, it is evident that the melanocyte dataset exhibited the lowest loss angle for the range of timescales studied. The averaged melanocytes loss angle peaked at just under 3°, and at a slightly higher frequency when compared to the fibroblasts (~5 kHz, plotted on the right-side axis). The melanoma showed a moderate loss angle, peaking at approximately 4.5° and remaining relatively constant for frequencies below 5 kHz, in contrast to its healthy counterparts, for which the loss angle dropped more sharply at low frequencies relative to its peak value. This indicates a uniquely heterogeneous response for melanoma, which could not be characterized using a purely elastic treatment like the Hertzian model analysis of Fig. 5. The averaged melanoma response also showcases a distinct bimodal curvature, further differentiating it from melanocytes and fibroblasts. Lastly, the fibroblasts showcased the largest viscous response peak for all cell lines near 3 kHz and 7° but fell below the average melanoma response at low frequencies. The shapes of the curves for both healthy cell lines were similar to one another, although the magnitudes of their responses were separated by approximately a factor of 2.

The proportion of viscous to elastic action for the three cell types studied fell far below that of the human lung epithelial cell fits to the PLR model, which exhibited monotonically increasing loss angles above 20° for a range of test frequencies below those acquired here. Clearly, since the cell types are different, it is not expected that the lung cell loss angle predictions should coincide with those of the adherent human skin cells. Nevertheless, it is insightful to compare the magnitude of the loss and storage moduli for different cell types. For both the A549 and BEAS-2B lung epithelial cell samples, the storage modulus increased nearly linearly with frequency from approximately 400 Pa to approximately 1.1 kPa, whereas the loss modulus increased with frequency, from just over 100 Pa to approximately 1.1 kPa in a manner similar to that depicted in the inset of Fig. 6. Both lung epithelial cell lines displayed nearly identical response, with loss and storage moduli on a similar order of magnitude across the frequency spectrum, but with only the loss modulus exhibiting nonlinear changes with frequency. This stands in contrast with our results presented here, for which the loss and storage moduli do not always monotonically increase or decrease, and in fact, can show specific regions of the frequency spectrum where viscous action peaks. As expected, the PLR results exhibit relatively few features in the data, aside from the nonlinear, monotonic increase in viscous action.

## Discussion

As depicted in Fig. 4, all measured human skin cell types appear relatively soft when deformed with a spherical probe at their nuclear region, ranging from approximately 200−1200 Pa in total stiffness, but their storage and loss moduli vary widely in both their absolute values and in the proportion of viscous to elastic action (Fig. 6). Furthermore, the 2D adherent metastatic melanoma sample exhibited a uniquely wide confidence interval for both moduli. All cell types exhibited their strongest viscous action near 10 kHz (Fig. 4b), and mostly displayed decreasing viscous action below and above those frequencies, with the melanoma and fibroblast datasets showing larger loss moduli than melanocytes (for the averaged response of all curves). These observations suggest that analysis of the frequency-dependent viscoelastic behavior of human skin cells could be used to unambiguously differentiate cancerous cells from their normal counterparts.

For all cell types, the proposed iterative methodology delivered a satisfactory fit of the force curves observed in the AFM experiments. However, there remained features that were not represented well, as discussed above for all three cell types. Several possible explanations for the reduced fit quality, especially for the melanoma dataset, include relatively deep indentations (> 1 µm) and the existence of a thick glycocalyx layer on the cell exterior. In regard to deep indentations, the Lee and Radok viscoelastic framework is defined for relatively small strains; however, a recent computational study found that classical contact mechanics models are robust with higher indentations yielding errors less than 5%^47^. In the case of large glycocalyx thickness, it has been shown that the extracellular brush-like layer on cancer cells can be over 1 µm^48^, therefore supporting the use of relatively higher indentations. Note that indeed the glycocalyx layer could introduce significant artifacts to our viscoelastic calculations; however, currently, there is no viscoelastic model capable of fully capturing the complexity of the material properties of cells and tissues. It is important to realize that the inability of the chosen mechanical model to reproduce such features indicates that the corresponding viscoelastic behaviors are excluded from the implied viscoelastic properties (Figs. 4 and 6). Furthermore, in the application of the methodology one realizes that there exist multiple parameter sets which can reproduce the normalized data to a similar degree of accuracy. This means that iteratively introducing new terms into the model, while limiting the parameter space to some extent, does not sufficiently isolate the global-optimum parameters representing the data. Since each of these parameter sets also corresponds to a different viscoelastic harmonic response, there remains uncertainty in the extracted viscoelastic properties. This is visualized in the exceptionally wide confidence intervals for the melanoma data. While the wide confidence intervals can be seen as a shortcoming of the method or the model, they do provide additional physical insight that complements the “best fit” result. For example, inspection of the melanoma storage modulus plot (blue line) in Fig. 4a suggests that the elastic response of the material is much less than that of the melanocytes. However, consideration of the confidence interval suggests that the material stiffness could vary appreciably over the frequency range and come quite close to the melanocyte response for high frequencies. Similarly, inspection of the corresponding loss modulus plot in Fig. 4b suggests that a softening behavior would be accompanied by a decrease in the loss modulus. Clearly, an important focus moving forward should be the inclusion of more sophisticated mechanical models, such as nonlinear viscoelastic models^49,50^, as well as the development of methods for further restricting the acceptable parameter space or for navigating the error surface of the fitting procedures with greater precision, in order to reduce the width of the confidence intervals. Ideally, the methodology should eventually capture all the small features present in the force and indentation data (within noise-related limitations) to maximize the possibility of discovering clear mechanical markers for specific biological conditions.

Despite many mechanical commonalities in cancer cells and tissues, subtle differences may exist in viscoelastic properties that may be critical to understanding tumor progression and metastasis in different situations. It is known that cancerous cells adapt to survive the harsh temporally evolving tumor microenvironment^51^, suggesting that many cellular properties, including mechanical properties, must also change accordingly. Several studies have shown that the mechanical properties of the tumor microenvironment play a critical role in cancer progression and metastasis, and have been correlated with the rate of cancer cell migration, proliferation, and resistance to chemotherapeutics^52^. In a tumor, the ECM is dynamically remodeled, and these modifications create a tumor microenvironment that is stiffer compared with the environment found in normal tissues. This promotes tumorigenesis through downstream signaling, thus forcing the cancer cells to modify their mechanical properties^53^. Previous studies have shown that most cancerous cells are softer than their non-malignant counterparts, and this correlates with their migration properties, whereby softer malignant cells are more migratory^18^. Interestingly, in melanoma cell lines it has been observed that early tumorigenic cells are softer than melanocytes, while highly metastatic melanoma cells were much stiffer than healthy melanocytes^54^. In a developing tumor, it is potentially more beneficial to have softer cells initially to allow more deformability and survival in the high pressure intratumoral microenvironment, whereas during metastasis a stiffer cell could be more beneficial to enable efficient migration throughout the tight confinements present in tissues. With this complexity in mind, it is reasonable to think that access to more physical parameters such as elasticity and viscosity at multiple timescales should provide much more information to aid the classification of cancer cells during disease progression rather than limiting the analysis to the use of only a deformation-rate-independent stiffness as a disease biomarker. For example, the loss angle has been shown to assist in identifying malignant and benign cell lines using information from only one frequency^37^, and providing a wider range of harmonic information could help enrich this approach.

The potential applications of the method described here are not limited to cancer. Other applications can be conceived, such as measurement of the viscoelastic properties at multiple timescales in the inner ear sensorial and nonsensorial tissues, in order to elucidate the role of tissue mechanical evolution in hearing loss. Recently, it has been shown that tensional homeostasis provided by non-muscle myosin II contractile activity in the mammalian cochlea is critical for hearing integrity^55^. Inhibition of myosin II contractile activity by Blebbistatin relaxes the organ of Corti and presumably softens it^55^. It has also been shown that TRIOBP activity reorganizes the filamentous cytoskeleton in sensory hair cells and supporting cells, thus TRIOBP is implicated in maintaining the mechanical properties of the organ of Corti, while loss of TRIOBP-5 isoform was shown to significantly decrease its apical surface stiffness^56^. Another potential application of our method is the characterization of artery walls with plaque buildups to more deeply understand the role of mechanics in atherosclerosis or other cardiovascular-based diseases. In atherosclerosis, arterial wall increased stiffness is associated with disease pathophysiology and cardiovascular risk events^57^. Notably, these studies and many others still characterize the mechanics of diseased systems by only measuring the elastic properties (stiffness), thus neglecting the rich viscoelastic behavior at different timescales, which as shown here, offers a much more unequivocal path for differentiating cells and tissues.

One key limitation of the current study is the range of timescales which can be accessed using standard quasi-static AFM force curves on live cells. At shorter timescales, the method is limited by the equipment data sampling speed, which can be alleviated by continued advancement in data acquisition rates. However, because the samples are living organisms, measuring their viscoelastic properties requires addressing a variety of complications including sample motion and a dynamic response to external inputs. Here, it was preferable to perform fast quasi-static force curves (at or below 1 s) to minimize the artifactual contributions of cell mechanosensing responses triggered by external mechanical stimuli, including cytoskeleton remodeling and morphological changes. It is critical to emphasize that the proposed methodology is capable of handling significantly longer timescale data sets, but the current test configuration limits the range of times that can be accessed, and by extension, the processes which can be observed.

In summary, we have introduced an iterative-fitting method to characterize the viscoelasticity of living cells at multiple timescales based on a simple-to-acquire input consisting of AFM quasi-static force curves. The study revealed that unique viscoelastic features emerging at different timescales can be used to precisely differentiate normal from cancerous cells. This approach could thus be exploited to categorize cells for disease diagnosis, monitoring, and treatment. Elucidating the complex viscoelastic behavior of living metastatic cells could also enable the engineering of novel therapeutic approaches designed to achieve an improved anti-tumor response. Therefore, we propose the use of viscoelastic properties at multiple timescales as a mechanical biomarker of diseases.

## Methods

### Cell culture and preparation

Human Foreskin Fibroblast cells were obtained from the American Type Culture Collection (ATCC, Cat #: SCRC-1041, Manassas, VA) and cultured in Dulbecco’s Modified Eagle’s Medium (DMEM, Life Technologies, Carlsbad, CA) supplemented with 10% fetal bovine serum (FBS, Life Technologies), 1 mM sodium pyruvate (Life Technologies), 1x GlutaMAX (Life Technologies), and 1% Penicillin-Streptomycin (Life Technologies). Human Primary Epidermal Melanocyte cells were obtained from ATCC (Cat #: PCS-200-013) and cultured in Dermal Cell Basal Medium (ATCC) supplemented with Phenol Red (ATCC) and Adult Melanocyte Growth Kit (ATCC). Human Melanoma A-375 cells (Cat #: CRL-1619) were obtained from ATCC and cultured in DMEM supplemented with 10% FBS (Life Technologies), 1× GlutaMAX (Life Technologies), 1X Antibiotic-Antimycotic (Life Technologies), and 20 mM HEPES pH 7.4.

Cells were plated on glass-bottom dishes (Willco Wells, Amsterdam, The Netherlands) to < 70% confluence. Cells were left to adhere on the glass bottom dishes overnight and maintained at 37 °C and 5% CO_2_. On the following day cells were transported to the AFM system and placed on the AFM X-Y stage to perform force spectroscopy AFM measurements.

### Atomic force microscopy

Live cell measurements were performed using a Bruker BioScope Catalyst AFM system (Bruker, Santa Barbara, CA) mounted on an inverted Axiovert 200 M microscope system (Carl Zeiss, Göttingen, Germany) equipped with a Confocal Laser Scanning Microscope 510 META (LSM 510 Meta, Carl Zeiss) and a 40× (0.95 NA, Plan-Apochromat) objective lens (Carl Zeiss). A Petri dish heating stage (Bruker) was used to maintain physiological temperature (37 °C) of cells during measurements. Modified AFM microcantilevers with an attached 25 µm-diameter polystyrene microsphere were obtained from Novascan (Novascan, Ames, IA). The AFM probe spring constant was obtained using the thermal tune method built into the AFM system. Calibrated spring constants for the cantilevers ranged from 0.5 to 1 N/m. After cantilever calibration, the AFM probe was placed on top of the nuclear region of an adherent cell. The deflection setpoint was set between 20 and 25 nm, yielding applied forces between 5 and 18 nN. The force curve ramp rate was set to 0.5 Hz and the probe speed ranged between 1.9 and 2.4 µm/s. Multiple consecutive quasi-static force curves were collected on each individual cell with a deflection trigger of 25 nm.

### Data analysis

To evaluate the performance of the iterative-fitting and previous open-search methodologies, a Windows 10 64-bit system with a 9th Generation Intel Core i9 processor and 32 Gb of RAM was used. The Matlab parallel processing toolbox function “parfor” was used to communicate the for-loop orders to each of eight total parallel workers. For every viscoelastic model configuration (i.e., 1-term, 2-term, etc.) 500 fitting attempts were made using the Generalized Maxwell and Generalized Kelvin−Voigt viscoelastic models (Supplementary Fig. 3) in the Lee and Radok indentation framework (Eq. S19).

For the data analysis of all cell types the Matlab script was modified to function on a High Performance Computing (HPC) cluster at the George Washington University (GWU). For the melanocytes and melanoma, standard computing nodes on the Pegasus HPC (Dell PowerEdge R740 server with Dual 20-core 3.70 GHz Intel Xeon Gold 6148 processors, 192 GB of 2666 MHz DDR4 ECC Register DRAM, 800 GB of onboard SSD storage, and Mellanox EDR Infiniband controllers to 100 GB fabric) were utilized to perform the same parallelized iterative fitting methodology and the augmented hardware specifications allowed 20 parallel workers to be used for each dataset. The increased processing power enabled a larger number of fitting attempts for each dataset. Specifically, 1000 fitting attempts based on random starting points were used for every model configuration. The processing time was dependent upon the length of the dataset under study but varied between 4 and 17 h. For the fibroblasts, due to time and computing cluster availability constraints at GWU, the National Institutes of Health (NIH) Biowulf cluster was used with a similar code repository (modified to meet specific HPC requirements for job scheduling). The 28-core “norm” queue for Biowulf (Dual 28-core × 2.4 GHz Intel E5-2680v4 processor, 256 GB of RAM, and a 56 Gb/s FDR Infiniband controller) was used for fitting, which allowed a maximum of 28 parallel workers. Due to a large number of cells included (33), the fibroblasts required approximately 147 h of total runtime on Biowulf.

The Matlab script used the “lsqcurvefit” (least squares) function with the “trust-region-reflective” internal algorithm selected. The cost function used was a simple Sum of the Squared Error (SSE) between the integral term in Supplementary Eqs. S4 and S5, and the normalized y-data (left-hand side) for both equations, respectively. The lsqcurvefit() function was chosen over other alternate nonlinear least-squares fitting functions because it showcased an increased speed of convergence compared to other options (specifically fmincon()), and the trust-region-reflective algorithm was used because it is the default for that function. Helpful information for benchmarking and troubleshooting new implementations of the viscoelastic parameterization approach outlined can be found in Supplementary Discussions 6 and 7.

To reproduce the curves shown in Figs. 4, 5, and 6, the reader is directed to Supplementary Discussion 8, which outlines the relationships used for creating the viscoelastic harmonic quantities and the pseudo-elastic model.

### Statistics and reproducibility

The viscoelastic results presented in this manuscript were calculated using the optimal parameter set obtained from analyzing the average of 70, 71, and 193 force curves from the melanoma, melanocyte, and fibroblast cell conditions, respectively. The averaging was performed in the frequency domain, after each optimal parameter set was used to calculate a predicted frequency-dependent curve for the storage modulus, loss modulus, and loss angle from every force curve. The solid lines in Figs. 4 and  6 are the simple average of these individual viscoelastic harmonic quantity predictions, and the confidence bands (shaded regions) are the range of observations for each discrete frequency using a 95% confidence level and the Student’s t distribution. In total, the averaged datasets contain curves collected from 13 unique melanoma cells, 12 unique melanocytes, and 33 unique fibroblasts.

Due to the intrinsic heterogeneity between individual cells and the complexity associated with recreating the exact conditions for force curve extraction using AFM, readers should not expect to recreate the experimental results exactly. Furthermore, the fitting process is moderately stochastic via the random starting point generated for each parameterization attempt—although this can be alleviated by using more fitting iterations. In the context of this manuscript, each force curve is considered a single experiment, and “replicates” are defined as the number of unique force curves considered for each type.

### Reporting summary

Further information on research design is available in the Nature Research Reporting Summary linked to this article.

## Supplementary information

Supplemental Information File

Description of Additional Supplementary Files

Supplementary Data 1

Reporting Summary

## Data Availability

The datasets for each cell type (melanocytes, melanoma, and fibroblasts) have been included in a github repository^58^, in the “data” directory. Due to size limitations, the output “.mat” files have been excluded from the repository—these can be made available upon reasonable request by contacting the authors. Any remaining information can be obtained from the corresponding author upon reasonable request. The source data for each figure has been provided in an excel document, labeled “Supplementary Data 1”.

## Code availability

The Matlab code used to acquire the optimal parameter sets obtained in this manuscript is available in a github repository^58^. In addition, the script used to create the manuscript figures is also included in the repository under the name “plotManuscriptFigures.m”.
