# Supplementary material for: Viscoelastic parameterization of human skin cells characterize material behavior at multiple timescales
Source: Commun Biol. 2022 Jan 11;5:17. doi: 10.1038/s42003-021-02959-5 (PMC8752830; doi:10.1038/s42003-021-02959-5)
Supplement: Supplementary file 1 — Supplemental Information File [file 42003_2021_2959_MOESM1_ESM.pdf]

# Supplementary Information For:

# Viscoelastic Parameterization of Human Skin Cells Characterize Material Behaviour at Multiple Timescales

Cameron H. Parvini[1], Alexander X. Cartagena-Rivera*[2], Santiago D. Solares*[1]

[1] Department of Mechanical and Aerospace Engineering, The George Washington University School of Engineering and Applied Science, Washington, District of Columbia, USA

[2] Section on Mechanobiology, National Institute of Biomedical Imaging and Bioengineering, National Institutes of Health, Bethesda, Maryland, USA

*Corresponding Authors: Alexander X. Cartagena-Rivera (alexander.cartagena-rivera@nih.gov); Santiago D. Solares (ssolares@gwu.edu)

## Supplementary Discussion 1: The Lee and Radok Framework for Spherical Indentation of a Viscoelastic Half-Space

To cast the problem of a spherical probe indenting a viscoelastic surface, it is necessary to make a series of simplifying assumptions about the tip-sample interaction. The longstanding geometry which Lee and Radok[1] proposed in 1960 applies for spherical, rigid indenters forced into a viscoelastic half-space, and is visualized in Supplementary Figure **2**.

A detailed breakdown of the derivation and associated assumptions for this framework has been provided previously[2], but it remains critical for users to confirm the validity of this approach for their specific conditions before implementing it. To that end, at least the following conditions must hold for the Lee and Radok derivation to be applicable:

1. The tip shape can be regarded as approximately spherical;
2. The indentation depth is sufficiently small, such that the strain behaves approximately linear;
3. The contact area is monotonically increasing during the experiment;
4. The problem can be cast as quasi-static (time is only a reference variable).

If the above can be assumed, the following relationship between applied load ($F$) and indentation depth ($h$) applies for a viscoelastic material having Poisson's ratio $\nu$ (commonly assumed to be incompressible, such that $\nu = 0.5$), and characteristic operators $u$ and $q$:

$$u[F(t)] = \frac{8\sqrt{R}}{3(1-\nu)} q[\{h(t)\}^{\frac{3}{2}}] \qquad \text{Eq. S1}$$

These characteristic operators are described as a polynomial series in the complex Laplace domain variable "s", and are related to the material Retardance ($U(s)$) and Relaxance ($Q(s)$) by:

$$U(s) = \frac{u(s)}{q(s)} = \frac{u_0 s^0 + u_1 s^1 + \cdots + u_n s^n}{q_0 s^0 + q_1 s^1 + \cdots + q_m s^m} \qquad \text{Eq. S2}$$

$$Q(s) = \frac{q(s)}{u(s)} = \frac{q_0 s^0 + q_1 s^1 + \cdots + q_m s^m}{u_0 s^0 + u_1 s^1 + \cdots + u_n s^n} \qquad \text{Eq. S3}$$

The Lee and Radok technique is particularly powerful for simulating tip-sample interactions in atomic force microscopy (AFM) because differential equations in the time domain can be cast as simple polynomials in the Laplace domain, provided that the viscoelastic model can be successfully described and transformed into the Laplace domain. Relatively straightforward rearrangement of Eq. S2 and Eq. S3 allows each order of the variable $s$ to be grouped, and the coefficients for each series can then be calculated using known material parameters. If either the force or indentation is provided, and the Lee and Radok assumptions still hold, calculating the material response simply requires taking discrete derivatives of the AFM observables.

Since the material models have been described using $U$ and $Q$, Eq. S1 is transformed into the Laplace domain and rearranged such that the indentation is the output variable. Thus:

$$\{h(t)\}^{\frac{3}{2}} = \frac{3(1-\nu)}{8\sqrt{R}}\,\mathcal{L}^{-1}\left[\frac{u(s)}{q(s)}F(s)\right] = \frac{3(1-\nu)}{8\sqrt{R}}\,\mathcal{L}^{-1}[\,U(s)F(s)]$$

$$\{h(t)\}^{\frac{3}{2}} = \frac{3(1-\nu)}{8\sqrt{R}}\int_0^t U(t-\zeta)F(\zeta)\,d\zeta \qquad \text{Eq. S4}$$

Here, the retardance of the Generalized Kelvin-Voigt model can replace the Retardance ($U$) in *Eq. S4*. Since the force and indentation are known throughout an AFM tip-sample interaction, the only unknown parameters are the compliances and characteristic times contained within $U$. This equation can also be easily re-written to utilize the Relaxance ($Q$) of the Generalized Maxwell Model:

$$F(t) = \frac{8\sqrt{R}}{3(1-\nu)}\int_0^t Q(t-\zeta)\{h(\zeta)\}^{\frac{3}{2}}\,d\zeta \qquad \text{Eq. S5}$$

**Supplementary Discussion 2: Guidelines for the Choice of a Viscoelastic Material Model**

For many problems in viscoelasticity, it is standard to assume that various combinations of springs and dashpots in series and/or parallel can reproduce the viscous and elastic material behavior observed during mechanical interactions. These descriptions are commonly known as “rheological” or “spring-dashpot” models, and usually fall into the broad category of “linear viscoelasticity”—a term used for models that assume the elastic and viscous action of a material can be analytically separated. These models are inherently limited in scope to small stresses or indentations and must be assembled into more complex configurations to successfully reproduce commonly observed material behaviors such as strain creep (increasing strain under applied constant stress) and stress relaxation (reduction in stress under applied constant strain). Intermediate combinations, such as the Standard Linear Solid (SLS) or Burgers models, still contain relatively few parameters for describing samples but can be useful for initial approximations or temporally limited datasets. For further discussion of these models, including the strengths and weaknesses of each, the reader is directed to the available literature[3,4].

Recently, Bonfanti et al. published an introduction on soft matter viscoelasticity, with emphasis on fractional power law models[5]. There also exist manuscripts by Efremov et al. which treat viscoelasticity in soft biological samples examined using AFM techniques, with an emphasis on parameter extraction[6] and on reviewing models applied in the literature[7]. However, all these publications focus little on utilizing models with a *discrete* number of retardation times to reproduce material behavior, in favor of power law models or the simpler intermediate combinations mentioned above. The benefit of fractional models, and other forms of continuous relaxation spectra approaches, is the lack of transition zones in the material response and the relatively small number of model elements required to describe material action across many timescales. While this is convenient for obtaining material property approximations with fewer parameters (thus reducing computational overhead and leading to simpler representations), the shape of the relaxation

spectra is dependent upon the number of terms. This means that if there is action on a timescale deemed interesting, it is difficult to separate and monitor that term because the power law model parameters control the shape of the response for the entire timescale spectrum. The issue is further exacerbated when many distinct features are present in a mechanical experiment dataset. In such a case, having discrete retardation times provides a distinct advantage, as one can observe the relative shift in characteristic time and stiffness of an element, which may correspond to specific markers in the harmonic response of the material, and differentiate between specific materials or, for example, biological material conditions, by monitoring changes in well-understood harmonic quantities such as the storage and loss moduli.

When dealing with cells, specifically, it is necessary to forego convenient, simplistic material models and provide a sufficiently large parameter set, such that complex material action linked to important timescales can be identified. The most straightforward implementation is a generalized viscoelastic model, with a dynamic (i.e., user-defined) number of discrete elements. As such, the critical contribution from this manuscript is a detailed outline of an iterative approach, which can provide important feedback for the user, and help to avoid both "overfitting" and improper extrapolation of the material response beyond reasonable limits. This methodology represents a significant step toward accurately characterizing materials where action can be linked to specific timescales and is necessary to support biological research efforts seeking new mechanical markers in cells.

The Generalized Kelvin-Voigt and Generalized Maxwell models are illustrated in Supplementary Figure **3**, along with their sub-components. Since they are conjugate models, they can both represent the same range of material action provided their Prony series are correctly parameterized[8]. However, it is commonly understood that the suitability of each model is dependent upon the problem boundary conditions. The Generalized Maxwell model is most easily described in terms of Relaxance (the material stress response to applied strain), whereas the Generalized Kelvin-Voigt model is conveniently formulated in terms of Retardance (strain response to applied stress)[3]. Approximate analytical relationships and numerical interconversion methods have been introduced to translate between the Prony series parameters of the two models[9].

These formulations can be considered the viscoelastic-counterparts to the Young's Modulus and Glassy Compliance of linear-elastic models. Whether elastic or viscoelastic, these quantities act as conversion factors between stress and strain. Unlike for the linear-elastic case where these operators are constant values, the viscoelastic Relaxance and Retardance are time-dependent.

Full derivations of the constitutive equations for each model pictured in Supplementary Figure **3** are available in the literature, which are based on the Laplace Transform[3,8] and fundamentals of continuum mechanics[10], but are beyond the scope of this appendix. As such, we begin by describing the viscoelastic Relaxance ($Q$) and Retardance ($U$) in terms of stress ($\sigma$) and strain ($\varepsilon$):

$$U(t) = \frac{\epsilon(t)}{\sigma(t)} \quad \text{Eq. S6}$$

$$Q(t) = \frac{\sigma(t)}{\epsilon(t)} \quad \text{Eq. S7}$$

These relationships are general and will need to be specified to the AFM static force spectroscopy (SFS) problem geometry later using one of the available indentation frameworks. For now, it will suffice to provide descriptions of each model using their appropriate boundary conditions. The Laplace Domain treatment of the mechanical descriptions in Supplementary Figure **3** is the simplest method for deriving relationships for $U$ and $Q$, using a technique analogous to the Electrical Impedance Method in circuit theory. Springs and dashpots are combined using common series and parallel rules to provide a single-element description—similar to a transfer function. As shown by Tschoegl[3], the Generalized Kelvin-Voigt model features the following Retardance:

$$U(s) = J_g + \Sigma_{i=1}^{N} \frac{J_i}{1+\tau_i s} + \frac{\phi_f}{s} \qquad \text{Eq. S8}$$

$$U(t) = J_g \delta(t) + \Sigma_{i=1}^{N} \frac{J_i}{\tau_i} e^{-\frac{t}{\tau_i}} + \phi_f \qquad \text{Eq. S9}$$

This description assumes that the model contains both a Glassy Compliance ( $J_g$ ) and a single steady-state fluidity ( $\phi_f$ ) in addition to N Voigt elements in series, each with their own compliance ( $J_i$ ) and characteristic time ( $\tau_i$ ). The Relaxance of a Generalized Maxwell solid can be similarly described as:

$$Q(s) = \frac{E_e \eta_f s}{E_e + \eta_f s} + \Sigma_{i=1}^{N} \frac{E_i \eta_i s}{E_i + \eta_i s} \qquad \text{Eq. S10}$$

$$Q(t) = E_g \delta(t) - \frac{E_e}{\tau_f} e^{-\frac{t}{\tau_f}} - \Sigma_{i=1}^{N} \frac{E_i}{\tau_i} e^{-\frac{t}{\tau_i}} \qquad \text{Eq. S11}$$

Here, the Elastic Modulus ( $E_e$ ), Glassy Modulus ($E_g = E_e + \Sigma_{i=1}^{N} E_i$), and steady state viscosity ( $\eta_f$ ) have been included in addition to the moduli ( $E_i$ ) and characteristic times ( $\tau_i$ ) of each Maxwell element up to N branches. Note that the damper viscosities have been replaced by the element characteristic times according to $\tau_i = \frac{\eta_i}{E_i}$ in Eq. S11. Additionally, in the case that the material is arrheodictic (i.e., it is unable to sustain steady-state flow), the second term in $Q(t)$ is excluded. This eliminates any relaxation of the model arm containing $E_e$, meaning that as time trends toward infinity the value of $Q(t)$ will approach $E_e$; conversely, if the material is known to be rheodictic, then the term is included and the Relaxance will tend toward zero at long timescales. A lack of steady-state fluidity in the Generalized Kelvin-Voigt model indicates that the final term in the series $U(s)$ is removed. The time-dependent nature of these relationships means that for each calculation the entire stress or strain history must be known, then convolved with the appropriate spectrum to recover its pair.

It is worthwhile to note that the existence of an elastic term and steady-state fluidity in the material is not always reasonable. Lopez et al. suggested that the fluidity could be neglected for shorter indentation experiments (on polymers), as the overall interaction time would be well under the threshold for shear flow[8]. While true for many polymers, biological samples are often much softer and more fluidic at standard operating temperatures and pressures. As such it is recommended to include at least a single steady-state fluidity term when considering biological samples, with the understanding that more complex treatments may be necessary, such as the inclusion of fluidities at multiple timescales. Similarly, the elastic term may be negligible for highly fluidic samples. For the Generalized Maxwell case, the idea that a static stiffness acts in parallel with the other elements may be violated when the samples are sufficiently soft, and a clear majority of the energy is dissipated by viscous action. If an elastic term is included under such conditions, one could erroneously attribute the stiffness of short timescales to the elastic term and the total dissipated energy would be underestimated, because the elastic energy is returned after unloading the sample. It should be noted that, for the adherent skin cell lines studied in the accompanying main manuscript, the steady-state fluidity was excluded for simplicity. The performance of the methodology on cells, while also utilizing new configurations of the generalized viscoelastic models in rheodictic form, remains to be investigated.

There are several critical points to keep in mind when applying the parameterization methods presented. First and foremost, the methodology does not naturally prevent “overfitting” a dataset; in this context, overfitting occurs when a user inserts a larger number of model terms than is necessary to acquire a closer approximation to the data. Iteratively introducing terms of increasing

timescale gives a clear indication of when the minimum number of terms necessary for a close estimate occurs, but users must be careful to include only orders of magnitude that can reasonably be justified as being truly present in the dataset. For example, the likelihood that an indentation occurring for 1 millisecond contains mechanical artifacts from timescales on the order of $10^0$ is low and including a characteristic time on that order could obscure the effects of lower order terms. To avoid this issue, the smallest number of terms possible that provide an adequate fit (as defined by the user) should be chosen, and iteratively introducing terms clearly indicates when that occurs.

In addition to controlling the overall number of terms, the upper and lower bounds for all parameters must be appropriately chosen to avoid poor fitting performance and violating the physical principles of the material. For example, fitting a Generalized Kelvin-Voigt model without restricting the individual compliances of each term to values above zero would allow terms to apply negative (downward) forces from within the material, which would be unreasonable for a repulsive indentation experiment (where the material resists the tip motion). While this case is clear for the stiffness terms, the characteristic times are slightly more complex to consider. It is not unreasonable to specify specific values for each characteristic time and allow the stiffnesses to vary within the bounds discussed above, although this approach makes an implicit assumption that those characteristic times exist within the material. In this case, the user has limited the parameter space and may be able to acquire results quickly and with good accuracy. However, by removing the characteristic times as a degree of freedom in the fitting, one may obscure valuable insight into the sample's mechanical nature. On the contrary, by allowing the characteristic times for each term to vary within some orders of magnitude, users leave open the possibility that a more natural combination of parameters can represent the material to a higher degree of accuracy at the cost of computational overhead. When action, whether it be hardening, softening, or otherwise, is known a-priori to occur within a sample on a specific timescale, users should consider including specific terms in the model description. Inevitably, the purpose of these parameterization efforts is to acquire a deeper understanding of the mechanical response of the sample and failing to leverage previous knowledge can lead to unnecessary additional time and effort.

In extrapolating material behavior using parameterized models, it is critical to acknowledge the limitations of the parameters obtained by fitting a discrete dataset. The sampling frequency determines the lower (shorter) bounds of the window within which the model parameters are useful—action on timescales smaller than the sampling frequency cannot be accurately resolved within the dataset, and as such, it is unknown whether the material action will be properly predicted for a different set of experimental conditions where tip velocities are significantly faster. For instance, an SFS-based parameter set would be difficult to use for estimating tapping-mode AFM indentations because the interaction times in the latter technique are orders of magnitude shorter. Furthermore, at smaller timescales the sensor noise floor could play a dominating role in the "perceived" forces, leading to large errors. Similarly, the total repulsive indentation time determines the upper bounds of applicability for the model parameters. It would be inappropriate to assume that mechanical action applied over timescales that are several orders of magnitude larger than the duration of force application can be adequately discerned from the smaller-timescale response measured. For capturing data during long experiments, tightly controlling external sources of noise and error (room temperature, humidity, vibration, instrument drift, etc.) would also become increasingly important, as these changes could erroneously materialize in the dataset as stress-induced material action.

It is worthwhile to discuss the quantities of merit that can be equated between unique experiments and samples. Specifically, leveraging individual terms within a parameterized series is an invalid form of comparison. This is because there exist a vast number of theoretically appropriate parameter sets that can recreate the mechanical action contained within a single indentation dataset. To illustrate this point, best-fit parameter sets can contain individual stiffnesses that appear to vary widely, disproportionate with the overall rigidity of the sample. Until a method for differentiating between valid and invalid optimal parameter sets can be developed, it remains inadvisable to utilize specific model terms independently. Using the predicted stiffness of all terms within the model together is more appropriate, as the fitting algorithm optimizes the entire series

simultaneously to reproduce the apparent force-indentation relationship. The storage modulus, loss modulus, loss angle, and retardance (or relaxance) provide the current best measures for comparison between individual experiments. Calculation of the viscoelastic harmonic quantities from a parameterized model has been covered in several previous publications[2,8].

Lastly, and most fundamentally, the assumptions presented during the framework derivations must remain valid throughout the duration of the experiments. In particular, the linearity assumptions dictate that the indentation depth must be "sufficiently small" such that the force curves appear approximately linear. While acceptable for hard materials and fast indentations, this condition could be easily violated for soft or biological samples. There is no specific numerical threshold for an indentation being too deep, although it is common to observe the indentation strain measure $\frac{l(t)}{R}$ and treat it similarly to the engineering strain quantity $\frac{\Delta L(t)}{L_0}$. The commonly referenced (though flexibly defined) transition from "linear strains" to "finite strains" then becomes applicable for the indentation frameworks presented, and strain values under 1-2% would normally ensure the linearity requirement for many common materials, depending on the material.

## Supplementary Discussion 3: Interconversion between the Generalized Kelvin-Voigt and Generalized Maxwell Models

It is known that the Generalized Maxwell and Generalized Kelvin-Voigt models are "mechanically equivalent" to one another. Analytically this is enforced by the definitions given above (Eq. S2 and Eq. S3), which reveal that the Relaxance ($Q(s)$) and Retardance ($U(s)$) are inverses of one another in Laplace space. Since the Generalized Maxwell and Generalized Kelvin-Voigt models can be written in terms of Relaxance and Retardance, given that the observed material action is the same in both cases, there must exist a set of parameters that satisfy the simple relation $Q(s)U(s) = 1$. This understanding is critical when experimental convenience demands implementation of one model over the other—the need for well-behaved system excitations (e.g., step-inputs) in stress or strain can require users to utilize a stiffness- or compliance-based model. In such situations, the user must understand that an appropriate parameterization of one model, given that it reproduces the input datasets to a high degree, will supply the parameters for its pair through one of several methods:

1. Using the extracted parameters to "simulate" the response to a step function in stress or strain using Eq. S4 or Eq. S5 and fitting the desired model to this new dataset;
2. If the models are simple enough (e.g., if they contain only a single element, such as for the SLS), analytical relationships exist for converting between parameters[3];
3. The Collocation Method, which involves calculating the complex s-domain distribution of one model and fitting the other model's parameters to the result.

If expediency is important, option 1 is convenient because it does not require conversion to the Laplace domain for either model (although this step is analytically straightforward). In many cases, option 2 is not feasible—beyond a single element, the relationship between models involves fourth order polynomials in the parameters and is ill-posed. Even for two elements, the mathematical treatment required would easily exceed the time or patience of most analysts.

The remaining option is to use the s-domain Collocation Method outlined by Tschoegl[3], which involves using the Laplace Domain forms of the Relaxance and Retardance (Eq. S8 and Eq. S10) with the understanding that $Q(s)U(s) = 1$. As such, evaluating $Q$ or $U$ for a wide range of s-values will show a shape similar to an inverted-logistic function with several features in the transition region. The user can then perform a fit using the inverse of the complementary spectra to obtain the parameters that create mechanical equivalence between the two models.

Finally, if the representation of a model in the harmonic space is desired, one need not perform a fit to obtain equivalent parameters before calculating the relevant harmonic quantities (loss modulus, loss compliance, storage modulus, storage compliance, loss angle). Instead, relationships exist that allow for conversion between these quantities, based on the use of the absolute modulus ($\tilde{E}$) and absolute compliance ( $\tilde{J}$):

$$E'(\omega) = \frac{J'(\omega)}{\tilde{J}(\omega)^2}$$ Eq. S12

$$E''(\omega) = \frac{J''(\omega)}{\tilde{J}(\omega)^2}$$ Eq. S13

$$J'(\omega) = \frac{E'(\omega)}{\tilde{E}(\omega)^2}$$ Eq. S14

$$J''(\omega) = \frac{E''(\omega)}{\tilde{E}(\omega)^2}$$ Eq. S15

Where the absolute modulus and compliance are calculated as:

$$\tilde{E}(\omega) = \left[\left(E'(\omega)\right)^2 + \left(E''(\omega)\right)^2\right]^{\frac{1}{2}}$$ Eq. S16

$$\tilde{J}(\omega) = \left[\left(J'(\omega)\right)^2 + \left(J''(\omega)\right)^2\right]^{\frac{1}{2}}$$ Eq. S17

Thus, if the desired output quantities are the loss modulus ($E''$) and storage modulus ($E'$) when the user has a parameterized Generalized Kelvin-Voigt model (i.e., with native loss compliance $J''$ and storage compliance $J'$), these relationships can be used to convert between the quantities successfully. This is possible because these distributions exist in the complex domain—conversion between time-domain quantities still requires fitting (e.g., $Q(t)$ to $U(t)$). For a full suite of the equations necessary to calculate the harmonic moduli, the reader is directed to the literature[2,3,8].

**Supplementary Discussion 4: Summary of Equations Required for Implementation of the Methodology**

With this understanding of the generalized models and indentation framework one can begin extracting parameters from properly conditioned AFM SFS datasets. The steps required for conditioning raw AFM observables for fitting have been previously enumerated[2] and are thus excluded here. The following equations are the only ones necessary to extract material information using the generalized viscoelastic models and the parameter extraction methodology:

$$\{h(t)\}^{\frac{3}{2}} = \frac{3(1-\nu)}{8\sqrt{R}} \int_0^t U(t-\zeta)F(\zeta)\,d\zeta$$ Eq. S4, Lee and Radok Spherical Indentation Framework for Force as the Input

$$F(t) = \frac{8\sqrt{R}}{3(1-\nu)} \int_0^t Q(t-\zeta)\{h(\zeta)\}^{\frac{3}{2}}\,d\zeta$$ Eq. S5, Lee and Radok Spherical Indentation Framework for Indentation as the Input

$$U(t) = J_g\delta(t) + \Sigma_{\mathrm{i=1}}^{N}\frac{J_i}{\tau_i}e^{-\frac{t}{\tau_i}} + \phi_f$$

Eq. S9, Generalized Kelvin-Voigt Model Relaxance

$$Q(t) = E_g\delta(t) - \frac{E_e}{\tau_f}\ e^{-\frac{t}{\tau_f}} - \Sigma_{\mathrm{i=1}}^{N}\frac{E_i}{\tau_i}\ \ e^{-\frac{t}{\tau_i}}$$

Eq. S11, Generalized Maxwell Model Retardance

**Supplementary Discussion 5: Data Simulation Methods**

To adequately characterize the performance of both iterative- and open-search methods, AFM SFS data was simulated using known viscoelastic parameters and a dynamically-generated differential equation series. Because the Lee and Radok method only applies rigorously for monotonically increasing contact area, only the approach portion of the SFS curve was simulated. As previously stated, the Laplace transform and viscoelastic operators $U$ and $Q$ can be used in conjunction with a viscoelastic model to create a polynomial representation of the material. By using an approach similar to the calculation of electrical impedances in a circuit, the material descriptions from Supplementary Figure **3** were reduced to Eq. S8 and Eq. S10. At this point, the series can be easily rearranged to have the same form as Eq. S2 and Eq. S3, using standard algebraic operations. However, as the number of terms included in the viscoelastic description increases, these polynomials become increasingly complex. Beyond two or three additional elements, the fourth order polynomials are onerous to derive manually. In this case, utilizing a symbolic computational toolbox (such as the Matlab Symbolic Toolbox, Wolfram Mathematica, or Python's SymPy) can more readily handle the necessary simplification, factorization, and re-organization. Providing Eq. S8 and Eq. S10 to a symbolic programming tool can thus dynamically generate the coefficients required to simulate the following well-known differential representation of viscoelastic materials:

$$\sum_{i=0}^{N} q_i \frac{\partial^i \epsilon(t)}{\partial t^i} = \sum_{j=0}^{M} u_j \frac{\partial^j \sigma(t)}{\partial t^j} \qquad \text{Eq. S18}$$

Here the variables M and N represent the highest order of s associated with the desired polynomial series, and are denoted as separate exclusively for the case where u(s) and q(s) do not contain the same order of s. The quantities $u_i$ and $q_i$ represent the coefficient for the $i^{th}$ order derivative of the stress and strain, respectively, which can be obtained by rearranging the polynomial series in the complex variable $s$, as described above. Importantly, when the Generalized Kelvin-Voigt representation utilizes a steady-state fluidity term, the polynomial will also feature an order of $s^{-1}$, which in the time domain represents integration from the onset of indentation to the current time. The stress and strain in Eq. S18 are then replaced by functions of force and indentation. The following relationship can be used to generate AFM force curve data for the Lee and Radok (Eq. S19) indentation framework:

$$\frac{8\sqrt{R}}{3(1-\nu)}\left[\sum_{i=0}^{N} q_i \frac{\partial^i \{h(t)^{\frac{3}{2}}\}}{\partial t^i}\right] = \sum_{j=0}^{M} u_j \frac{\partial^j F(t)}{\partial t^j} \qquad \text{Eq. S19}$$

Once the coefficients $u_i$ and $q_i$ are available, one can simulate the tip motion in time by programmatically beginning with the AFM probe located at an initial height, and the enforcing an approach velocity towards the surface for the cantilever base. Each timestep requires solving one or more standard kinematic equations of motion for the AFM cantilever, depending on the number of normal modes included in the cantilever model. In addition, discrete derivatives to the $N^{th}$ order are necessary at each timestep, such that Eq. S18 can be rewritten to solve for the highest derivative of the force. Using the strain and strain rates for the current timestep, in addition to the

force and force rates (below the highest order) from the previous iteration, one can calculate the highest derivative of the force for the current time. This value can then be discretely integrated to provide all force terms at the current time, including the lowest-order term corresponding to the tip-sample force. The resulting force can be added to any long-range tip-sample force model (e.g., to account for nonbonded forces such as van der Waals attraction, etc.) and fed forward to the next timestep as input into the tip acceleration. The selected timestep must be small enough to ensure stability of the simulation. Using a timestep on the order of $10^{-10}$ seconds for the tip-sample contact portion of the force curve is small enough to prevent divergence in most cases. If the user wishes to adjust the timestep for their own simulation, taking the period of their cantilever's primary mode (the inverse of the cantilever natural frequency) and selecting a timestep several orders of magnitude lower (at least 4) is an adequate starting point. For these simulations, the intended outcome was an evaluation of the computation time, memory requirements, and accuracy of the methodology.

The cantilever properties used for the simulation were chosen to match a compliant silicon-cantilever colloidal probe. The tip radius was chosen to be 12.5 µm, with the tip attached to a cantilever of stiffness 0.6 N/m, with a fundamental frequency of 75 kHz and quality factor of 2. The sample was approached at 2 µm/s, starting from 1 µm away. The long-range tip-sample forces were not included, and indentation continued until a trigger force of 5 nN was achieved. The surface was given an elastic modulus of 9220 Pa, a Poisson's Ratio of 0.5, and steady-state fluidity was excluded. The Prony series parameters used are provided in Table S1.

**Supplementary Discussion 6: Comments on Convergence and Benchmarking**

For a critical portion of the process, the outlined viscoelastic parameter extraction methodology relies heavily upon accurate numerical approximations of the data. As such, the issues encountered cease to be analytically centered, and instead involve the programming implementation of those relations. Determining "convergence" and establishing well-behaved benchmarks for performance are important steps to validating that the implementation does, indeed, provide the insight that is claimed by the user.

On the subject of convergence, there are several quantities that merit discussion. First and foremost is the number of fitting attempts made. This is defined here as the number of unique iterations where the algorithm of choice is given the model description, output data, and a starting point for each parameter. To converge to a global minimum error with the current methodology, it is necessary to attempt a sufficiently large number of unique starting parameter combinations, such that the parameter space is widely explored. This clearly incurs computational overhead but is necessary because the final parameters are very sensitive to their initialization values. If the number of iterations is too low, then the likelihood is diminished that the model was provided enough opportunity to achieve good convergence. Instead, the number of iterations must be sufficiently high that parameter sets on their way to high-fidelity approximations can fully converge to a solution. However, allowing too many iterations can also lead to poor performance, especially when the optimization algorithm encounters a plateau in the parameter space. Such plateau regions can be large and may have no discernable negative gradients. In this case the algorithm will take small steps and simply run out of iterations without making any significant progress. If the allowed number of iterations is too large the algorithm can waste considerable amounts of time attempting to converge a poorly conditioned parameter set.

Benchmarking the performance of the programming implementation usually requires processing known (or well characterized) datasets and evaluating the fitting quality and resources used against an established approach. In the case of viscoelastic parameter extraction, this would usually involve using a simulated dataset with known parameters to which the methodology is applied. The user can then assess how well the results match the known parameters of the model and decide whether that level of accuracy is enough for their application. It can also be beneficial to simulate datasets that resemble the expected experimental data in both timescale and order of magnitude. This allows a priori optimization of the number of iterations, fitting attempts and range of randomized input parameters. Clearly, measurement noise and other factors will influence the process when using real data, but selecting a tighter range of randomized starting points and optimizing the parameter choices can speed up convergence.

## Supplementary Discussion 7: Comments on Common Troubleshooting Methods

Most often, performance issues can be identified under a few categories (in order of decreasing frequency, in our experience):

1. Settings for the fitting attempt (not allowing enough iterations, using a poorly chosen range for initial guesses, etc.);
2. Outliers in the dataset (e.g., when averaging curves, for example, keeping one or more datasets which showcase highly erratic material action due to measurement instabilities);
3. Coding implementation (programming bugs, introduction of error due to discrete approximations, poorly chosen algorithms, functions with default settings that cause confusion, etc.);
4. Analytical implementation (issues with the derivation of the equations as implemented in the code).

Of all these categories, the coding implementation errors can be the most time consuming to fix. It usually is not immediately clear when a coding error is causing the problem, which reinforces the need to run common benchmarking operations *before* evaluating real data. Provided the simulated data is well understood, this can showcase programming issues more quickly. Building in simple-to-activate feedback (e.g., plots, status messages, etc.) can also speed up this process, at the expense of initial complexity.

If changing the number of allowed iterations or fit attempts does not rectify the issue under consideration, is necessary to begin by confirming that the viscoelastic models appear to act as expected in the code. Often, small issues like referencing the viscoelastic parameters in an incorrect order can cause confusing intermediate outputs which may not be immediately obvious. Checking that any subfunctions are working properly, are up-to-date, and any old versions are deleted or out of memory usually follows.

Lastly, and anecdotally frequent, are hidden settings in standard functions. For example: the convolution functions in Matlab and Python generally default to a "full" setting. This means that the output vector from the convolution of two vectors with length N and M respectively will be N+M-1 in length. If this is not taken into account, inappropriate clipping of the data may occur. Behaviors of functions that are unexpected, or worse, *change* with new distributions of the coding environment, can be difficult-to-identify sources of error. As before, it remains important to build in safeguards that notify the user when data is ill-conditioned or unexpected data manipulations are being performed.

## Supplementary Discussion 8: Reproducing the Viscoelastic Harmonic Moduli Shown in the Main Manuscript

To successfully reproduce the viscoelastic harmonic moduli plots shown in the main manuscript, it is necessary for one to have a parameterized viscoelastic model which has been optimized against either the observed force or scaled indentation via Eq. S4 or Eq. S5 depending upon the model. Once the parameter set has been acquired, the storage and loss modulus can be calculated for the Generalized Maxwell model by:

$$E'(\omega) = E_g - \sum_{i=1}^{n} \frac{E_i}{1+\tau_i^2\omega^2} \qquad \text{Eq. S20}$$

$$E''(\omega) = \sum_{i=1}^{n} \frac{E_i\tau_i\omega}{1+\tau_i^2\omega^2} \qquad \text{Eq. S21}$$

Alternatively, for the Generalized Kelvin-Voigt, the storage and loss compliance take the form:

$$J'(\omega) = J_g + \sum_{i=1}^{n} \frac{J_i}{1 + \tau_i^2 \omega^2} \quad \text{Eq. S22}$$

$$J''(\omega) = \sum_{i=1}^{n} \frac{J_i \tau_i \omega}{1 + \tau_i^2 \omega^2} \quad \text{Eq. S23}$$

Note that these quantities can be interconverted by using the equations outlined in Section 3 above. Recall that Fig. 4 and 6 from the main manuscript have been created by calculating $E'$ and $E''$ for every individual curve on each cell type, and then subsequently averaged in the frequency domain to acquire the solid lines representing the mean response predicted by all experiments. The confidence intervals are the 95% CI using the Student's t distribution. As described in the main manuscript, the loss angle is the inverse tangent of the loss modulus over the storage modulus (or, alternately the loss compliance over the storage compliance).

Lastly, the pseudo-elastic elastic modulus plotted in Fig. 5 was similarly optimized against the observed force for each individual curve from all cell types. The relationship is far simpler than those presented above:

$$F(t) = \frac{4\sqrt{R}}{3(1-\nu^2)} E_e h^{\frac{3}{2}} \quad \text{Eq. S24}$$

Instead of a series of parameters, the pseudo-hertzian relationship contains only a single stiffness $E_e$ and known constants and quantities ($R$, $\nu$). Note that here, and everywhere else in the manuscript, Poisson's ratio is assumed to be 0.5 (i.e. the material is assumed to be incompressible). After minimizing the fit error to find $E_e$, one has a distribution of stiffnesses predicted by each curve and can perform the statistical analyses outlined in the main manuscript to acquire Fig. 5a and plot the optimal model prediction using Eq. S24 for each cell type.

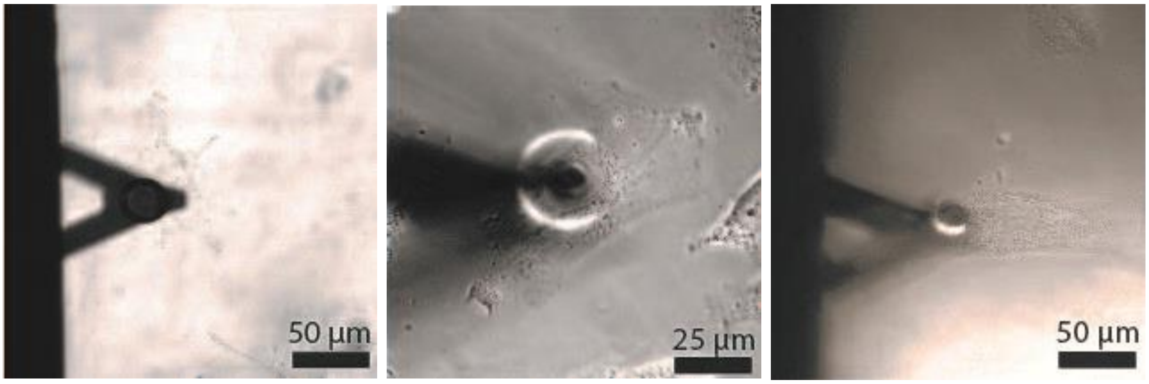


(a) (b) (c)

**Supplementary Figure 1: Phase Contrast Light Microscopy Images for Each Adherent Skin Cell Type under Study.** This figure shows phase contrast light microscopy images for a 2D adherent human primary epidermal melanocyte cell (a), human metastatic melanoma A375 cell (b), and human foreskin fibroblast (c).

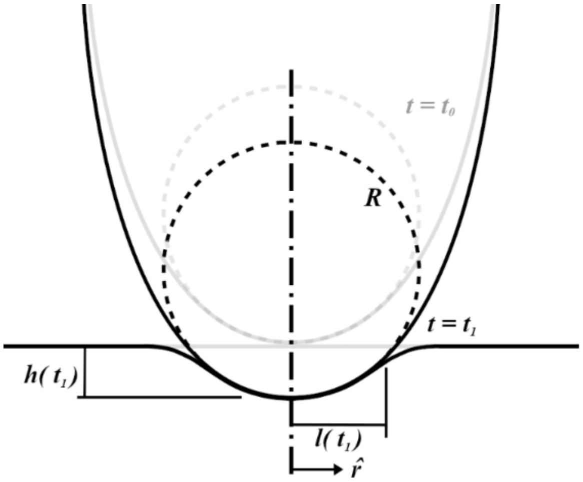


**Supplementary Figure 2: Spherical Indentation Geometry Proposed by Lee and Radok[1].** The deepest indentation occurs at the sphere’s central axis (r = 0) and is labeled as h(t); the indenter has a radius of curvature R; the distance from the central axis to the edge of contact is labeled as l(t) and is also commonly referred to as a(t).

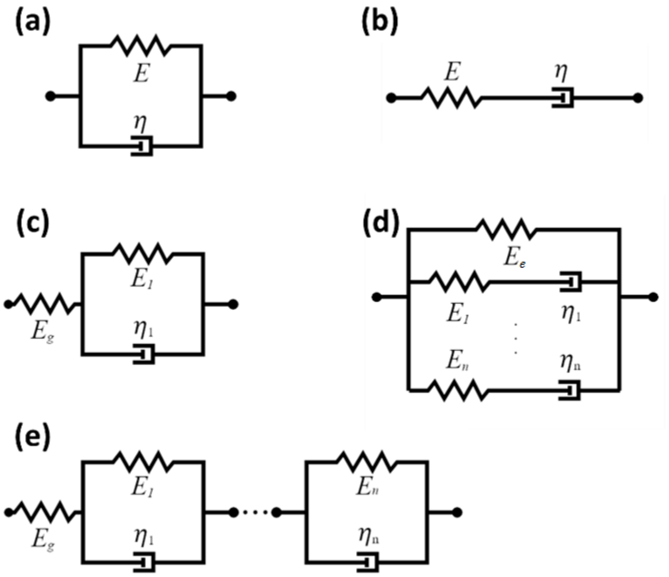


**Supplementary Figure 3: Common Viscoelastic Models.** Viscoelastic descriptions are fundamentally comprised of spring (elastic) and damper (viscous dissipative) elements; (a) and (b) are the Kelvin-Voigt and Maxwell elements respectively; (c) is the Standard Linear Solid (SLS) in the Kelvin-Voigt configuration; lastly, (d) and (e) are the generalized models discussed in this manuscript, the Generalized Maxwell and Generalized Kelvin-Voigt, respectively. Note that the SLS model can also be configured to use a Maxwell element, wherein (b) is placed in parallel with a spring. As is discussed in this appendix, the Maxwell and Kelvin-Voigt models are mechanically equivalent and can be interconverted, although the conversion is not always simple when handling the generalized forms (c & e).

**Supplementary Table 1. Viscoelastic Prony Series Parameters Used for Data Simulation**

| Model Element Number | Stiffness (Pa) | Characteristic Time (s) |
|---|---|---|
| 1 | 6691 | 6.84E-4 |
| 2 | 1877 | 4.40E-3 |
| 3 | 9684 | 2.97E-4 |

**Supplementary Table 2. Standard Error of the Regression for the Data Visualizations Presented in Figures 2, 3, and 5 of the Main Manuscript**

| Figure 2 | | | |
|---|---|---|---|
| Subfigure Label | Number of Elements | Color | Error* |
| a | 1 | Red | 6.869e-08 |
| | 2 | Blue | 3.721e-08 |
| | 3 | Green | 1.226e-07 |
| | 4 | Magenta | 5.296e-08 |
| c | 1 | Red | 2.004e-09 |
| | 2 | Blue | 2.134e-09 |
| | 3 | Green | 2.486e-09 |
| | 4 | Magenta | 2.954e-09 |
| Figure 3 | | | |
| b | 2 | Red | 3.200e-10 |
| | 3 | Blue | 6.380e-10 |
| | 2 | Green | 3.283e-10 |
| c | 1 | Red | 1.311e-07 |
| | 2 | Blue | 1.697e-08 |
| | 3 | Green | 1.698e-08 |
| | 4 | Magenta | 4.659e-08 |
| | 5 | Black | 3.610e-08 |
| d | 1 | Red | 6.533e-08 |
| | 2 | Blue | 3.405e-08 |
| | 3 | Green | 3.384e-08 |
| | 4 | Magenta | 3.467e-08 |
| | 5 | Black | 3.385e-08 |
| e | 1 | Red | 8.510e-08 |
| | 2 | Blue | 1.741e-08 |
| | 3 | Green | 1.875e-08 |
| | 4 | Magenta | 1.703e-08 |
| | 5 | Black | 1.815e-08 |
| Figure 5 | | | |
| b | | Red | 3.200e-10 |
| | | Blue | 6.374e-10 |
| | | Green | 2.315e-10 |

* Note: The error used is the Standard Error of the Regression, defined as $S = \sqrt{\frac{SSE}{N-2}}$, where SSE denotes the Sum of Squared Errors.

**Supplementary References**


1. Lee, E. H. & Radok, J. R. M. The Contact Problem for Viscoelastic Bodies. *Journal of Applied Mechanics* **27**, 438-444 (1960).
2. Parvini, C. H., Saadi, M. A. S. R. & Solares, S. D. Extracting viscoelastic material parameters using an atomic force microscope and static force spectroscopy. *Beilstein Journal of Nanotechnology* **11**, 922-937 (2020).
3. Tschoegl, N. W. *The Phenomenological Theory of Linear Viscoelastic Behavior: An Introduction*. (Springer-Verlag Berlin Heidelberg, 1989).
4. López-Guerra, E. A. & Solares, S. D. Modeling viscoelasticity through spring–dashpot models in intermittent-contact atomic force microscopy. *Beilstein J. Nanotechnology* **5**, 2149-2163 (2014).
5. Bonfanti, A., Kaplan, J. L., Charras, G. & Kabla, A. Fractional viscoelastic models for power-law materials. *Soft Matter* (2020).
6. Efremov, Y. M., Wang, W.-H., Hardy, S. D., Geahlen, R. L. & Raman, A. Measuring nanoscale viscoelastic parameters of cells directly from AFM force-displacement curves. *Scientific Reports* **7**, 1541 (2017).
7. Efremov, Y. M., Okajima, T. & Raman, A. Measuring viscoelasticity of soft biological samples using atomic force microscopy. *Soft Matter* **16**, 64-81 (2020).
8. López-Guerra, E. A., Eslami, B. & Solares, S. D. Calculation of standard viscoelastic responses with multiple retardation times through analysis of static force spectroscopy AFM data. *Journal of Polymer Science Part B: Polymer Physics* **55**, 804-813 (2017).
9. Park, S. W. & Schapery, R. Methods of Interconversion Between Linear Viscoelastic Material Functions. Part I - A Numerical Method Based on Prony Series. *International Journal of Solids and Structures* **36**, 1653-1675 (1998).
10. Eringen, A. C. in *Mechanics of Continua*, 318-400 (John Wiley & Sons Inc., 1967).
