# Supplementary material for: Viscoelastic parameterization of human skin cells characterize material behavior at multiple timescales
Source: Commun Biol. 2022 Jan 11;5:17. doi: 10.1038/s42003-021-02959-5 (PMC8752830; doi:10.1038/s42003-021-02959-5)
Supplement: Supplementary file 2 — Description of Additional Supplementary Files [file 42003_2021_2959_MOESM2_ESM.pdf]

Description of Additional Supplementary Files

File name: Supplementary Data 1

Description: The data underlying each figure and subfigure is presented in this workbook; sheets exist for each figure, and the datasets are grouped according to subfigure.
