# Supplementary material for: Viscoelastic parameterization of human skin cells characterize material behavior at multiple timescales
Source: Commun Biol. 2022 Jan 11;5:17. doi: 10.1038/s42003-021-02959-5 (PMC8752830; doi:10.1038/s42003-021-02959-5)
Supplement: Supplementary file 4 — Reporting Summary [file 42003_2021_2959_MOESM4_ESM.pdf]

nature portfolio

Corresponding author(s): Alexander X. Cartagena-Rivera, Santiago D. Solares

Last updated by author(s): Nov 15, 2021

Reporting Summary

Nature Portfolio wishes to improve the reproducibility of the work that we publish. This form provides structure for consistency and transparency in reporting. For further information on Nature Portfolio policies, see our Editorial Policies and the Editorial Policy Checklist.

Statistics

For all statistical analyses, confirm that the following items are present in the figure legend, table legend, main text, or Methods section.

n/a	Confirmed	
☐	☒	The exact sample size (*n*) for each experimental group/condition, given as a discrete number and unit of measurement
☐	☒	A statement on whether measurements were taken from distinct samples or whether the same sample was measured repeatedly
☐	☒	The statistical test(s) used AND whether they are one- or two-sided<br>*Only common tests should be described solely by name; describe more complex techniques in the Methods section.*
☒	☐	A description of all covariates tested
☐	☒	A description of any assumptions or corrections, such as tests of normality and adjustment for multiple comparisons
☐	☒	A full description of the statistical parameters including central tendency (e.g. means) or other basic estimates (e.g. regression coefficient) AND variation (e.g. standard deviation) or associated estimates of uncertainty (e.g. confidence intervals)
☒	☐	For null hypothesis testing, the test statistic (e.g. *F*, *t*, *r*) with confidence intervals, effect sizes, degrees of freedom and *P* value noted<br>*Give P values as exact values whenever suitable.*
☒	☐	For Bayesian analysis, information on the choice of priors and Markov chain Monte Carlo settings
☒	☐	For hierarchical and complex designs, identification of the appropriate level for tests and full reporting of outcomes
☒	☐	Estimates of effect sizes (e.g. Cohen's *d*, Pearson's *r*), indicating how they were calculated

Our web collection on statistics for biologists contains articles on many of the points above.

Software and code

Policy information about availability of computer code

Data collection	NanoScope 9.1
Data analysis	Matlab 2019b

For manuscripts utilizing custom algorithms or software that are central to the research but not yet described in published literature, software must be made available to editors and reviewers. We strongly encourage code deposition in a community repository (e.g. GitHub). See the Nature Portfolio guidelines for submitting code & software for further information.

Data

Policy information about availability of data

All manuscripts must include a data availability statement. This statement should provide the following information, where applicable:

- Accession codes, unique identifiers, or web links for publicly available datasets
- A description of any restrictions on data availability
- For clinical datasets or third party data, please ensure that the statement adheres to our policy

The AFM force curve datasets for each cell type (melanocytes, melanoma, and fibroblasts) have been included in a public github repository, in the "data" directory.

Field-specific reporting

Life sciences study design

All studies must disclose on these points even when the disclosure is negative.

Sample size	Three unique cell types were examined for their bulk, time-dependent viscoelastic response. The results of this manuscript were calculated using 70, 71, and 193 independent force curves from melanocytes, melanoma, and fibroblasts respectively. These curves were collected from 13 unique melanoma cells, 12 unique melanocytes, and 33 unique fibroblasts, and the force curves are organized in the online repository according to which cell they were captured from.
Data exclusions	It is common in Atomic Force Microscopy analysis to remove force curves which feature excessive noise or sudden jumps in force or indentation correlating to surface inhomogeneities (voids, tip sliding, etc.). For this manuscript a total of 6 curves were ignored for the melanoma cells, 14 curves were removed from the melanocyte population, and 7 were excluded for the fibroblasts.
Replication	Due to the intrinsic heterogeneity between individual cells and the complexity associated with recreating the exact conditions for force curve extraction using AFM, readers should not expect to recreate the experimental results exactly. Furthermore, the fitting process is moderately stochastic via the random starting points generated for each parameterization attempt---although this can be alleviated by using more fitting iterations. In the context of this manuscript, each force curve is considered a single experiment and "replicates" are defined as the number of unique force curves considered for each type. By these definitions we collected a significant number of force curves from unique cells, and the population results should give an indication of the overall viscoelastic response for each cell type.
Randomization	The groups were not randomly allocated because cells of the same type had to be cultured together. Cells from different areas of each dish (a total of two dishes for each cell type) were used to collect the force curves, introducing a sufficient level of randomness for the current study.
Blinding	Blinding was not relevant to this study because the force curves were not corrected by the users directly (only via standard code for all types), and the viscoelastic inversion process is inherently stochastic and optimization-based. Neither process requires user input to directly generate the data---curves are processed from raw readings collected by the AFM sensors, and the same inversion code is used for all cell types. The process is thus inherently independent from the investigators until after the data has been analyzed and plotted.

Reporting for specific materials, systems and methods

We require information from authors about some types of materials, experimental systems and methods used in many studies. Here, indicate whether each material, system or method listed is relevant to your study. If you are not sure if a list item applies to your research, read the appropriate section before selecting a response.

Materials & experimental systems

n/a	Involved in the study
☒	☐ Antibodies
☒	☐ Eukaryotic cell lines
☒	☐ Palaeontology and archaeology
☒	☐ Animals and other organisms
☒	☐ Human research participants
☒	☐ Clinical data
☒	☐ Dual use research of concern

Methods

n/a	Involved in the study
☒	☐ ChIP-seq
☒	☐ Flow cytometry
☒	☐ MRI-based neuroimaging
